# Natural Products Modulating Angiotensin Converting Enzyme 2 (ACE2) as Potential COVID-19 Therapies

**DOI:** 10.3389/fphar.2021.629935

**Published:** 2021-05-03

**Authors:** Murtala Bello Abubakar, Dawoud Usman, Gaber El-Saber Batiha, Natália Cruz-Martins, Ibrahim Malami, Kasimu Ghandi Ibrahim, Bilyaminu Abubakar, Muhammad Bashir Bello, Aliyu Muhammad, Siew Hua Gan, Aliyu Ibrahim Dabai, M Alblihed, Arabinda Ghosh, Reem H. Badr, Devarajan Thangadurai, Mustapha Umar Imam

**Affiliations:** ^1^ Department of Physiology, Faculty of Basic Medical Sciences, College of Health Sciences, Usmanu Danfodiyo University, Sokoto, Nigeria; ^2^ Centre for Advanced Medical Research and Training, Usmanu Danfodiyo University, Sokoto, Nigeria; ^3^ Department of Pharmacology and Therapeutics, Faculty of Veterinary Medicine, Damanhour University, Damanhour, Egypt; ^4^ Faculty of Medicine, University of Porto, Porto, Portugal; ^5^ Institute for Research and Innovation in Health (i3S), University of Porto, Porto, Portugal; ^6^ Laboratory of Neuropsychophysiology, Faculty of Psychology and Education Sciences, University of Porto, Porto, Portugal; ^7^ Department of Pharmacognosy and Ethnopharmacy, Faculty of Pharmaceutical Sciences, Usmanu Danfodiyo University, Sokoto, Nigeria; ^8^ Department of Pharmacology and Toxicology, Faculty of Pharmaceutical Sciences, Usmanu Danfodiyo University, Sokoto, Nigeria; ^9^ Department of Veterinary Microbiology, Faculty of Veterinary Medicine, Usmanu Danfodiyo University, Sokoto, Nigeria; ^10^ Department of Biochemistry, Faculty of Life Sciences, Ahmadu Bello University, Zaria, Kaduna Sate, Nigeria; ^11^ School of Pharmacy, Monash University Malaysia, Bandar Sunway, Malaysia; ^12^ Department of Microbiology, Usmanu Danfodiyo University, Sokoto, Nigeria; ^13^ Department of Microbiology, College of Medicine, Taif University, Taif, Saudi Arabia; ^14^ Microbiology Division, Department of Botany, Gauhati University, Guwahati, India; ^15^ Department of Botany and Microbiology, Faculty of Science, Alexandria University, Alexandria, Egypt; ^16^ Department of Botany, Karnatak University, Dharwad, India; ^17^ Department of Medical Biochemistry, Faculty of Basic Medical Sciences, College of Health Sciences, Usmanu Danfodiyo University, Sokoto, Nigeria

**Keywords:** medicinal plants, renin-angiotensin-system, COVID-19, ACE2, SARS-CoV-2.

## Abstract

The 2019 coronavirus disease (COVID-19) is a potentially fatal multisystemic infection caused by the severe acute respiratory syndrome coronavirus-2 (SARS-CoV-2). Currently, viable therapeutic options that are cost effective, safe and readily available are desired, but lacking. Nevertheless, the pandemic is noticeably of lesser burden in African and Asian regions, where the use of traditional herbs predominates, with such relationship warranting a closer look at ethnomedicine. From a molecular viewpoint, the interaction of SARS-CoV-2 with angiotensin converting enzyme 2 (ACE2) is the crucial first phase of COVID-19 pathogenesis. Here, we review plants with medicinal properties which may be implicated in mitigation of viral invasion either via direct or indirect modulation of ACE2 activity to ameliorate COVID-19. Selected ethnomedicinal plants containing bioactive compounds which may prevent and mitigate the fusion and entry of the SARS-CoV-2 by modulating ACE2-associated up and downstream events are highlighted. Through further experimentation, these plants could be supported for ethnobotanical use and the phytomedicinal ligands could be potentially developed into single or combined preventive therapeutics for COVID-19. This will benefit researchers actively looking for solutions from plant bioresources and help lessen the burden of COVID-19 across the globe.

## Introduction

By the end of December 2020, less than a year after it was declared an outbreak, the severe acute respiratory syndrome coronavirus-2 (SARS-CoV-2) had infected over 80 million people and claimed almost two million lives (European Center for Disease Prevention and Control, 2020). This places its mortality score over a thousand-fold greater than that of its homolog, the 2002/2003 SARS-CoV (WHO, 2004; de Wit et al., 2016). To date (30th, January. 2021), based on a MEDLINE search, due to its severity, over 90,000 papers (and possibly more) have addressed the 2019 coronavirus disease (COVID-19). COVID-19 develops after SARS-CoV-2 gains entry into host cells by binding to angiotensin converting enzyme 2 (ACE2) receptors (Hoffmann et al., 2020). This crucial first step initiates a cascade of events that results in a vicious cycle of virion to cell infection and replication that includes attachment, penetration, uncoating, translation, replication, assembly and release. The fusion to ACE2 receptor and internalization causes down-regulation of the ACE2 receptor, halting its traditional protective functions over the renin-angiotensin system (RAS) and potentiating the pathophysiological sequelae of COVID-19 (Verdecchia et al., 2020).

In this way, finding solutions to COVID-19 is at the core of solving this global pandemic. At present, available pharmaceuticals face numerous limitations in the treatment of COVID-19. The challenges range from safety and side effects to poor efficacy which warrants the search for better treatment modalities (Gupta and Misra, 2020; Nittari et al., 2020). Like with SARS-CoV, persistent complications, such as lung damage, make preventive strategies the best option (Kuba et al., 2005). In this pursuit, the development of vaccines and therapeutic approaches conceptually linked to ACE2 rank high for preventing COVID-19 (Bourgonje et al., 2020). Vaccines are either undergoing trials (with some concerns) or facing issues of distribution, acceptance or storage, and are a long shot from herd immunity. Meanwhile, in the search for ACE2-centered therapeutics, scientists have uncovered an ACE2 receptor decoy called human recombinant ACE2 (hrsACE2; APN01) (Monteil et al., 2020). These synthetic approaches may prove expensive and may take longer to verify across people and conditions.

**FIGURE 1:**
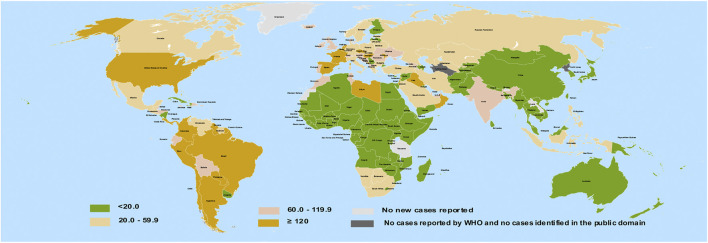
Geographical distribution of COVID-19 infection per 100,000 of population across 14-days. Redrawn from source: European Center for disease Prevention and Control (ECDC). Data for September 26, 2020 (European Center for disease Prevention and Control, 2020).

Natural products with some degree of biological benefits are regarded as bioactive compounds. Phytochemicals include a broad spectrum of plant bioactive compounds which are majorly proven to be safe. In recent times, these phytochemicals obtained from herbs have gained prominence in ethnomedicine as attractive choices for antiviral therapies (Utomo et al., 2020). Some polyphenols, a subset of phytochemicals, have been suggested to inhibit the fusion and entry of SARS-CoV-2 (Paraiso et al., 2020). So far, reports indicate that most African countries bear lesser COVID-19 burden compared to some Northern and Western nations of the globe (Figure 1). To explain this, several reasons have been proposed, including demography, climate, genetic variations, cross immunity, and antimalarial usage (Lalaoui et al., 2020).

**FIGURE 2:**
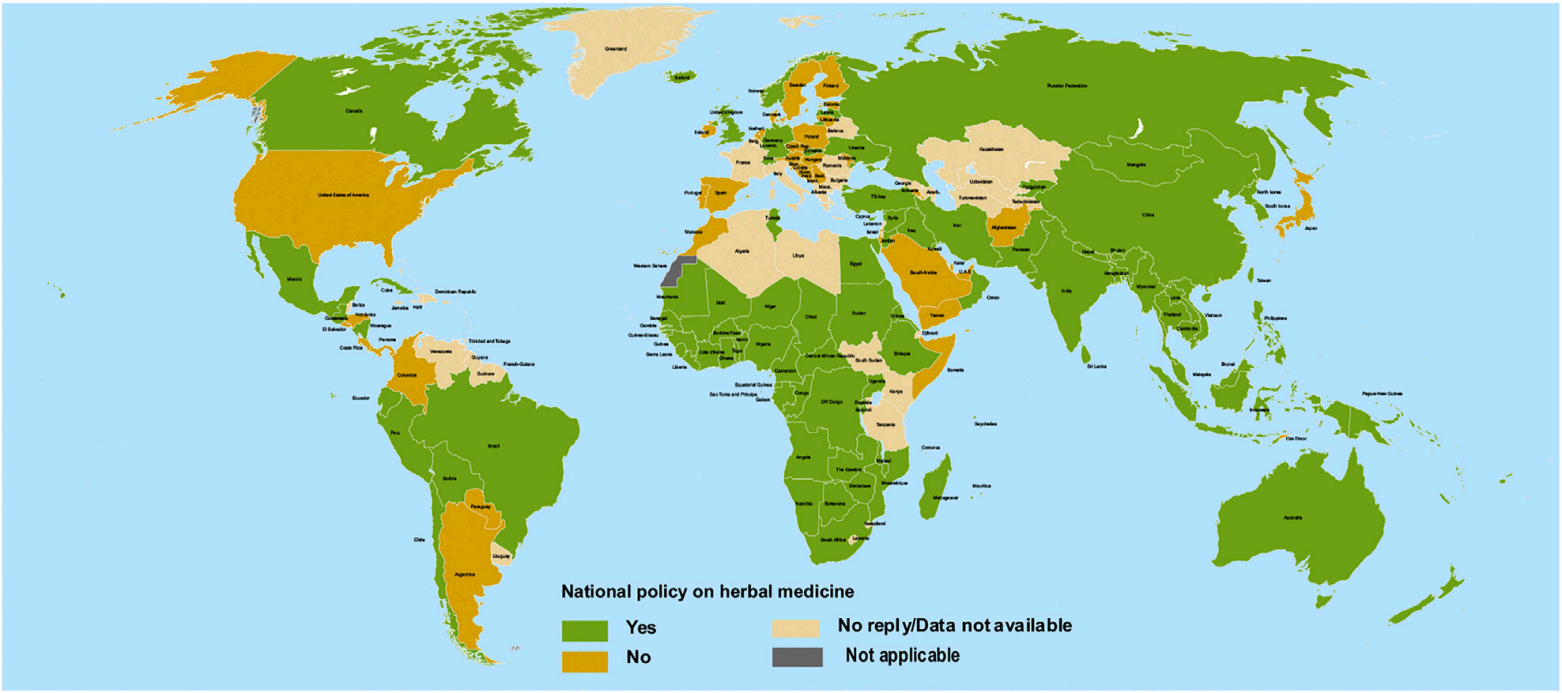
Geographical national level policy for herbal medicines among 194 countries. Redrawn from source: WHO Global Report on Traditional and Complimentary Medicine (WHO, 2019).

The African and Asian regions are famous for their ethnomedicinal herbs (Figure 2) and a close comparison reveals a previously unseen correlation between countries having no ethnomedicinal policies and a higher burden of COVID-19 (compare Figures 1, 2). Ethnomedicinal policies are developed following the demanding use of ethnomedicines. In turn, these policies offer goals and objectives to governments for the development and support of ethnomedicines and may include guideline principles related to safety and efficacy (Oyebode et al., 2016; WHO, 2019). The uniform rise in incidence of COVID-19 in regions of lower ethnomedicinal policies hints to a possible link between ethnomedicinal use and COVID-19 incidence. Despite the number of reasons put forward to explain the relatively mild emergence of COVID-19 in regions like Africa compared to America and Europe (Lalaoui et al., 2020), no involvement of ethnomedicinal use has been suggested until now. Possibly, ethnomedicinal plants contain bioactive compounds that contribute to prevent or mitigate SARS-CoV-2 invasion via interactions with ACE2-mediated viral tropism (fusion and entry).

Given the global burden and challenge posed by the pandemic and the urgent need for the rapid development of efficient, safe, cost-effective, and readily available prevention and treatment modalities, we turned to phytomedicine. Following the spike of converging evidences, we sought to review evidences related to ACE2 that may suggest the use of phytochemical remedies. Here, we also take a closer look at the SARS-CoV-2 and the indirect phytomedicinal targeting of ACE2 mediated tropism. This aims to provide an indirect evidence for the use of ACE2 interactive phytochemicals, singly or combined, for preventing and mitigating COVID-19, its symptoms and clinical co-morbidities. Notably, the influence of ethnomedicines on ACE2 modulation is not an exclusive factor in determining the rate of spread of COVID-19, but a highlight that identifies what logical next steps can be developed based on existing research. This will benefit investigators in quest for remedies from phytomedicine.

### Morphology and Genomic Organization of SARS-CoV-2

**FIGURE 3:**
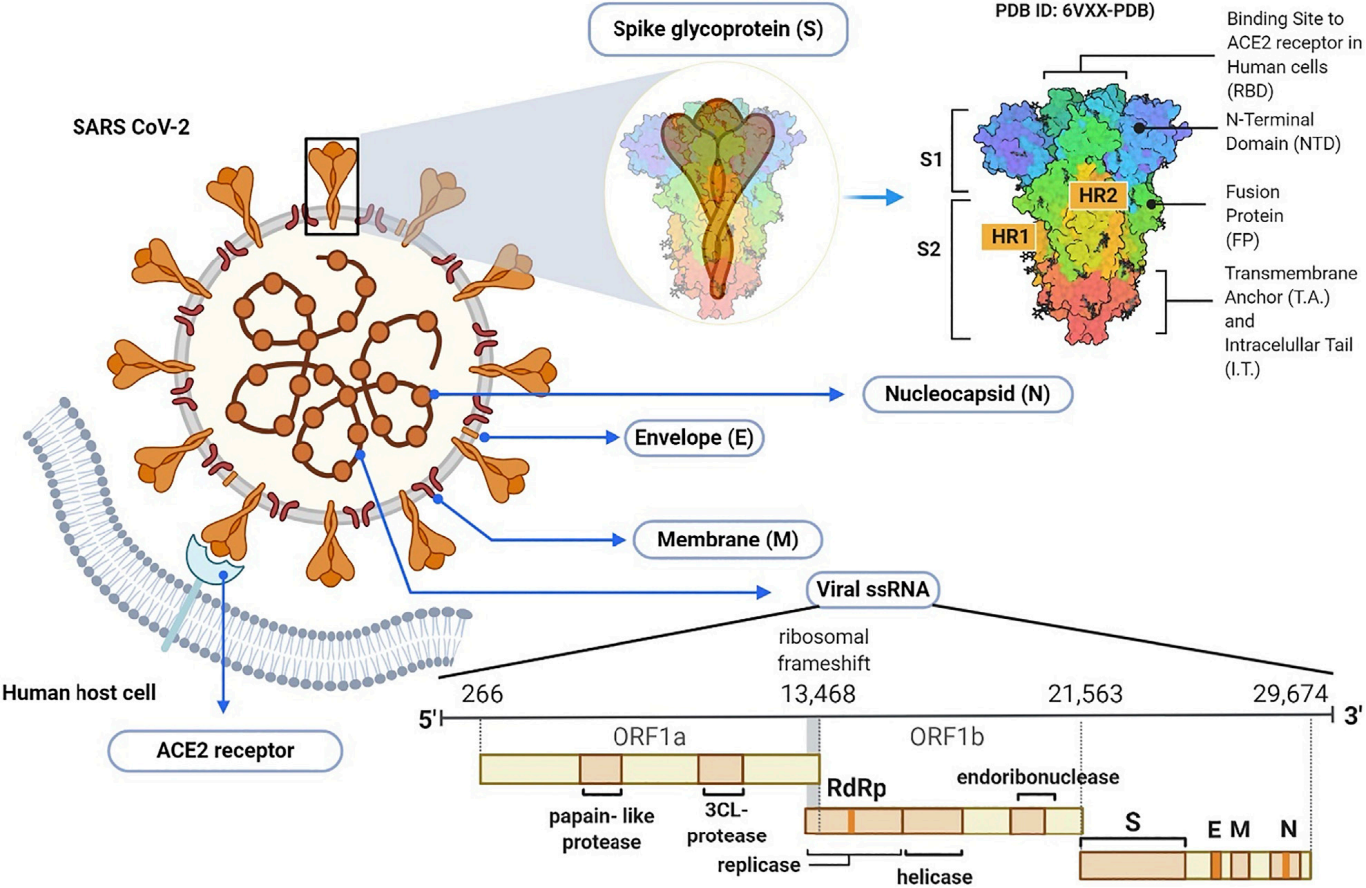
Schematic illustration of SARS-CoV-2 virion and its genome.

Coronaviruses are taxonomically grouped into the order of Nidovirales, family Coronaviridae and subfamily Orthocoronavirinae. The subfamily is further divided into alpha, beta, delta, and gamma coronavirus genera. The beta coronavirus genus comprises SARS-CoV, MERS-CoV and the recently emerged SARS-CoV-2 (Chen et al., 2020c). Morphologically, all coronaviruses are enveloped with crown-like particles enclosing a positive sense RNA genome (Figure 3). The lipid bilayer envelope which surrounds the viral particle is derived from the host cell membrane, and embeds three structural proteins, namely: 1) membrane (M), 2) envelope (E), and spike (S) glycoproteins. The other viral structural protein, nucleoprotein (N), is internally located and intimately associated with the viral genomic RNA (Kumar et al., 2020b).

The SARS-CoV-2 genomic RNA has approximately 30 kb in size and is made up of 13–15 (12 functional) open reading frames (ORFs) that are arranged in 5–3′ order of appearance. The first ORF from the 5′ end comprises approximately 67% of the genome and encodes several non-structural proteins. On the other hand, the remaining ORFs encode both accessory and structural proteins, including the major S, E, M, and N proteins (Naqvi et al., 2020). The M and E proteins are required for virus morphogenesis, assembly and budding, while the S glycoprotein is a fusion viral protein comprised of two subunits; S1 and S2. The S1 subunit is made up of signal peptide, N-terminal domain (NTD) and a receptor-binding domain (RBD) (Watanabe et al., 2020). The S2 subunit contains two heptad-repeat regions known as HR-N and HR-C, which form the coiled-coil structures surrounded by the protein ectodomain. Importantly, the S protein in its native form is coated with polysaccharide molecules, a property that partly enables SARS-CoV-2 to evade surveillance of the host immune system during entry (Shah et al., 2020).

### Pathophysiology of COVID-19

Although coronaviruses target the respiratory tract, they can also affect the gastrointestinal (GI) tract, kidney, central nervous (CNS) and cardiovascular (CV) systems (Peiris et al., 2003; To et al., 2004). Their systemic effects manifest as lesions and multiple organ dysfunctions especially in immunocompromized patients and the elderly with already existing history of chronic ailments like cancer, CNS disorders, and diabetes. Similarly, the organs that are predominantly affected by SARS-CoV-2 express high levels of ACE2, meaning that such organs, due to ACE2, have high affinity to SARS-CoV-2 reception (Letko et al., 2020). The ACE2 can catalyze the hydrolysis of angiotensin II (a vasoconstrictor peptide) into angiotensin (1–7) which is a vasodilator that lowers the blood pressure (Verdecchia et al., 2020). Eventually, the invasion of SARS-CoV-2 deviates the ACE2 functionality, causing increase in blood pressure, disruption of blood vessels, and inflammation of organs (Letko et al., 2020).

Notably, the SARS-CoV-2 preferentially targets the respiratory tract. Alveolar type II cells (AT2) of the lungs express high levels of ACE2, making them the primal targets of SARS-CoV-2 (Verdecchia et al., 2020). In the native alveoli, oxygen is essentially absorbed, whereas carbon (IV) oxide is released. However, due to the presence of SARS-CoV-2, ACE2 is prevented from modulating the protein angiotensin II (ANG II), thereby causing further damage to the blood vessels of the lungs, overall preventing oxygen and carbon (IV) oxide exchange. The resultant effect is difficulty in breathing and improper functioning of the lungs, which worsen the damaging effect to the lung parenchyma, ending up in bronchitis, edema, alveolar collapse and acute respiratory distress syndrome (Huang et al., 2020; Tan et al., 2021).

Should the invasion continue, the virus may gradually infiltrate and infect the circulating immune cells which carry it to other organs like the digestive tract, (i.e. stomach, duodenum, colon, and ileum) displaying inflammatory changes by attacking the epithelial cells of the mucous membrane of ileum and colon (Shi et al., 2005). In a broad view, ACE2 is expressed in abundance within the small intestine. Autopsy under high-resolution electron microscopy has shown that the virus replicates rapidly in intestinal tissue, thereby preventing the ACE2 from carrying out its functions in amino-acid balance, intestinal inflammatory response, homeostasis of the intestinal microbiota and regulatory expression of antimicrobial peptides. Consequently, a secondary infection by bacteria may also affect the weakened intestine, since ACE2 has been hindered from regulating the intestinal microbiota (Wang et al., 2020a). Due to low movement of bowels, an early sign of digestive tract invasion by SARS-CoV-2 could be diarrhea.

Circulating coronavirus in the blood stream can migrate to the heart and affect the cardiac tissue (Hamming et al., 2004). Accordingly, underlying cardiac injury elevates the risk of disease severity in COVID-19 due to heightened risks of myocarditis (To et al., 2004; Chen et al., 2020a). Kidney dysfunction is also a common feature of COVID-19 (Li et al., 2020c), as evidenced by reports of increased blood urea nitrogen, serum creatine and urea (Fan et al., 2020; Yang et al., 2020b). Urinary system failure, genital invasion and possibly death are also possible outcomes (Fan et al., 2020; Zhang et al., 2020b).

The most detrimental of all possibilities is damage to the CNS, when the SARS-CoV-2 nucleic acid contaminates the cerebrospinal fluid of the spinal cord and brain stem. Using the neuronal pathway, i.e., motor and sensory neurons, it streams via the nerves of the olfactory lobe of the nasal cavity to the brain. In fact, the use of motor proteins, kinesins and dynein, prominent to these neuronal pathways is essential for viral migration. The olfactory lobe, a part of the brain that translates smell responses, is the starting point of dysfunction. This partly explains the early signs of inability to smell, i.e. anosmia (Sun and Guan, 2020; Wu et al., 2020). Additionally, since the brain stem controls vital functions including blood pressure, heart beat and reflex actions like respiration, viral perforation of the brain stem eventually results in multiple organ dysfunctions. The development of intracranial infection may result in confusion, headache and epilepsy. Also, more serious complications, such as obstruction of cerebral blood flow, brain swelling, and coma could also occur. These are mostly mediated by interleukins (such as IL-6, IL-12, IL-15) and tumour-necrosis factor (TNF) in the brain, indicating an inflammatory response (Li et al., 2020b; Wu et al., 2020).

Notably, several organs could also deviate from proper functioning, such as the spleen and liver, but those highlighted above are the most susceptible. Furthermore, aside factors such as patient’s age, patient’s immune system, early diagnosis and treatment, the level of ACE2 expression also affects the COVID-19 pathophysiology. These pathophysiological features of COVID-19 as highlighted, could be quite detrimental and chronic; therefore, necessitating the need for more research into effective therapies for the prevention and/or treatment of COVID-19.

### COVID-19 and ACE2

One of the key signaling pathways that acts as a homeostatic regulator for the cardiovascular system is the RAS. Irrespective of an individual’s health status, RAS maintains a dynamic control of vascular function. This is achieved through different regulatory components and effector peptides such as the carboxypeptidase, ACE2, which converts angiotensin II to a vasodilator, angiotensin (1–7) through the ACE2/Ang one to seven axis (Donoghue et al., 2000; Tikellis and Thomas, 2012). ACE2 counteracts the effect of ACE/RAS pathway (de Kloet et al., 2010). ACE2 is an integral membrane protein present in the lungs, liver, heart, kidney, and endothelium (Donoghue et al., 2000). More importantly, ACE2 receptors are abundant in the epithelial cells of the surfaces of the nostrils, mouth, and lungs (Chen et al., 2020b). Sequence analysis demonstrated that ACE and ACE2 have over a 40% homology of their respective amino acids (Donoghue et al., 2000). The 40 kb ACE2 gene located on chromosome Xp22 consists of 18 exons and 20 introns and codes for a protein of 805 amino acids (Turner et al., 2002; Marian, 2013).

Unlike other coronaviruses that explore several receptors, like aminopeptidase N and dipeptidyl peptidase four in addition to ACE2 in gaining entry into cells during infection, SARS-CoV-2 exploits only the ACE2 protein for cell entry and subsequent viral replication (Zhou et al., 2020). The N-terminal region of the ACE2 receptor is anchored by the spike glycoprotein (S1) of SARS-CoV-2. Following receptor binding, SARS-CoV-2 employs some of the host proteases for spike protein priming. These include cathepsin L, cathepsin B, trypsin, factor X, elastase, furin, and transmembrane protease serine 2 (TMPRSS2) (Gheblawi et al., 2020). Being the major point-of-entry for SARS-CoV-2, the ACE2 protein expression on respiratory epithelial cell surfaces is critical for the pathogenesis of COVID-19 in individuals exposed to the virus. The vulnerability of the lungs to SARS-CoV-2 infection is premised on two facts; the large surface area of the lungs and the vast expression of ACE2 proteins on the type 2 pneumocytes of the alveoli in the lungs (Zhao et al., 2020). It is also noteworthy to state that the GI tract, especially the small intestine and colon could be critical points of entry since previous studies have demonstrated a high expression of ACE2 in these organs (Hamming et al., 2004; Li et al., 2020a). Also, the multiple organ failure in individuals with complicated COVID-19 could be linked to the vast distribution of ACE2 receptors in these organs (Guan et al., 2020; Huang et al., 2020) indicating the important roles of ACE2.

### ACE2 as a Therapeutic Target

Though, a focus on the ACE2 activity accompanying COVID-19 may be considered commonplace, it is arguably one of the significant direct/indirect targets for therapeutics in COVID-19. ACE2 physiology is important in hypertension, cardiac, kidney, and lung physiology (Tikellis and Thomas, 2012). The apparent relevance of ACE2 physiology in COVID-19 has come in two ways, both supporting immense clinical focus on its modulation: 1**)** as a receptor for SARS COV2 (ClinicalTrials.gov identifiers: NCT04335136) and 2**)** as an enzyme for the generation of ANG 1–7 (ClinicalTrials.gov identifiers: NCT04311177, NCT04312009, NCT04338009, NCT04338009, NCT04394117, and NCT04394117) (Bhalla et al., 2020). The unfortunate neglect of ACE2 therapeutic potential (and lessons learned) following SARS-CoV outbreak strongly necessitated and still advocates for the recent efforts to develop ACE2-centered therapeutics for the current SARS COV2 (Bhalla et al., 2020). Thus, efforts (till date) to find an ACE2-centered therapeutic are re-emerging with a steep focus after its rediscovery as a receptor for SARS COV 2 (Hoffmann et al., 2020).

To date, ACE2 receptor is the major known entry point for SARS-CoV-2 into human cells. The virus uses its spike protein in attaching to ACE2 receptor of susceptible cells. This would mean that a COVID-19 vaccine could be developed based on the spike protein sub-unit of SARS-CoV-2. In this regard, Tan and Colleagues have recently described a new vaccine candidate immunogenic against SARS-CoV-2 spike RBD, with demonstrated stability at ambient temperature; reducing cold-chain dependence (Tan et al., 2021). Alternatively, the development of small molecules or antibodies that are competitive antagonists for ACE2 receptor have been explored (Glasgow et al., 2020). These agents have shown superior neutralizing efficiency to convalescent sera (Glasgow et al., 2020; Tan et al., 2021).

Furthermore, Kuba and colleagues demonstrated that a downregulation of ACE2 protein by SARS-CoV leads to severe lung injury in laboratory mice (Kuba et al., 2005). By implication, this could mean that excessive administration of soluble ACE2 proteins would not only counteract the scenario of cell viral entry but also maintain the physiological activity of ACE2, including protection against lung injury via negative RAS regulation (Imai et al., 2005; Yu et al., 2016). This is the basis for recombinant ACE2 decoys (Monteil et al., 2020). Based on the clinical trials done so far, ACE2 protein that is synthesized through recombinant DNA technology, has shown to be efficacy in healthy and ARDS patients (Haschke et al., 2013; Khan et al., 2017). Recombinant ACE2 sequesters circulating viruses, thereby preventing the interaction between SARS-CoV-2 spike and endogenous ACE2 proteins. This effect allows endogenous ACE2 protein to negatively regulate the RAS, thereby preventing tissue injury as seen in COVID-19 (Zhang et al., 2020a). Notably, the oral delivery of ACE2 and Ang-(1–7) bioencapsulated in plant cells has been explored in non-COVID-19 disease states and may represent an innovative therapeutic strategy for COVID-19 (Shil et al., 2014). Additionally, the administration of Ang one to seven receptor agonists, like AVE 0991, has also been demonstrated to exert cardio-renal and pulmonary protective effects (Gheblawi et al., 2020).

Despite these efforts, the current absence of any approved ACE2-centered therapeutic (to reduce SARS-CoV-2 transmission, COVID-19 progression or halt cardiovascular complications) hints to the need for research in this respect (Bhalla et al., 2020). This is the reason why recent investigations are still ongoing toward developing an ACE2 interactive therapeutic (Esparza et al., 2020; Yahalom-Ronen et al., 2020) and much more brainstorming is needed.

### Natural Products and ACE2

Natural products have for long been considered as important sources of therapeutic agents. These agents are remarkably low molecular weight molecules capable of eliciting enzyme activities due to their complex nature (Malami et al., 2016). The secondary bioactive agents/plant metabolites, which are able to reduce or wholly inhibit enzyme catalytic activities, can be used as ACE2 modulators. Available evidence from previous studies supports the inhibitory properties of natural products toward ACE2 enzyme activity. Moreover, these natural products are considerably used in traditional medicine and are extensively found as part of the human diet. Here, we describe some of the common natural products that have been previously reported with ACE2 modulatory activity prior to the COVID-19 era (Table 1).

**TABLE 1:** Some natural bioactive compounds shown to modulate ACE2 prior to the COVID-19 era.

Natural products	Bioactive compound	Method of assessment	References
Flavonoids	Naringenin apigenin	*In vivo*	(Sui et al., 2010; Wang et al., 2019)
	Baicalin	*In vivo*	(Wei et al., 2015)
Steroids and steroids glycosides	Ginsenoside Rg3	*In vivo*	(Liu et al., 2019)
Coumarins	Osthole	*In vivo*	(Shi et al., 2013; Hao and Liu, 2016)
Alkaloids	Nicotianamine	*In vitro*	(Takahashi et al., 2015)
	Emodin	*In vitro*	(Ho et al., 2007)

Flavonoids are widely found in a variety of fruits and vegetables, including citrus fruits and tomatoes (Tutunchi et al., 2020). Among the different classes of natural products combined, flavonoids represent the largest group of ACE2 inhibitors. In fact, several studies have previously implicated some flavonoids to possess inhibitory properties against ACE2 activity. For instance, a previous *in vivo* study showed that apigenin upregulates the expression of the ACE2 gene in spontaneously hypertensive rats (Sui et al., 2010). Wei and co-workers also demonstrated that baicalin, a natural flavone, attenuates angiotensin-II induced endothelial dysfunction by modulating ACE2 expression both at mRNA and protein levels (Wei et al., 2015). Naringenin, a flavanone found in grape fruit, can also attenuate hypertensive reno-vascular damage *in vivo* by downregulating the ACE2 expression (Wang et al., 2019). These reports highlight a potential of flavonoids in mitigating SARS-CoV-2 viral infection via modulation of ACE2. Notably, as is highlighted later in this review, flavonoids like naringenin, naringin, nobiletin, hesperidin, hesperetin, neo-hesperidin, pinocembrin, quercetin, myricetin, and kaempferol have shown potential ACE2 binding affinity toward the residues that contact S protein of SARS-CoV-2 (Chen and Du, 2020; Cheng et al., 2020; Güler et al., 2020; Ngwa et al., 2020; Omar et al., 2020; Yang et al., 2020a).

Another class of natural products with ACE2 modulatory properties are the steroids and steroid glycosides. Ginsenosides are naturally occurring steroid glycosides commonly found in ginseng rhizome. An *in vivo* study demonstrated that ginsenoside Rg3, a tetracyclic triterpenoid saponin, induces the upregulation of ACE2 levels and attenuates Ang II-mediated renal injury (Liu et al., 2019). Following the outbreak of SARS-CoV-2, a recent virtual screening revealed that the steroid, arundoin, and some steroid glycosides (azukisaponin I, 20(S),24(R)-ocotillol and ginsenoside Rg6) can modulate ACE2 activity (Zi et al., 2020). In this study, the bioactive compounds demonstrated promising activity with a lower percentage inhibition based on an ACE2 kinase inhibition assay. Additionally, glycyrrhizin, another steroid glycoside found in the roots *Glycyrrhiza glabra* L., has been predicted to latch on to ACE2 with an estimated binding energy of −9 kcal mol^−1^, potentially preventing viral entry (Chen and Du, 2020).

Furthermore, coumarins, which are naturally occurring phenolic compounds, have also shown ACE2 modulatory properties. An *in vitro* and *in vivo* study showed that the prenylated coumarin, osthole, mitigates inflammation and acute lung injury in mice by preventing ACE2 and Ang (1–7) down-regulation, resulting in a lowered release of proinflammatory cytokines (TNF-α and IL-6) (Shi et al., 2013). This protective influence of osthole was proposed to occur due to the counter effects ACE2/Ang (1–7) has over the ACE/AngII axis, and was confirmed by an abrogation effect following the use of an ACE2 inhibitor (Shi et al., 2013). In a related study, Hao and Liu demonstrated that osthole attenuates pulmonary fibrosis and inhibits lung inflammation by modulating ACE2 in rat models of bleomycin-induced pulmonary fibrosis (Hao and Liu, 2016).

With regards to alkaloids, a recent study has highlighted that cepharanthine, a naturally occurring alkaloid¸ can bind to spike protein and inhibit the viral interaction with ACE2 protein *in silico* (Ohashi et al., 2020). In another study, the non-proteinogenic alkaloid, nicotianamine, exhibited a similar effect on ACE2 (Chen and Du, 2020), which agrees with a previous *in vitro* demonstration by Takahashi et al. (2015). Also, evidence had shown that a natural anthraquinone, emodin, significantly inhibits the infectivity of S protein-pseudotyped retrovirus to Vero E6 cells by binding to ACE2 protein and blocking the SARS-CoV S protein from binding to ACE2 at a 50% minimum inhibitory concentration (IC_50_) of 200 μM (Ho et al., 2007).

### Functional Foods and ACE2

Apart from providing nutrients and energy, functional foods also modulate the body’s physiological functions (Nicoletti, 2012). The potentially numerous health benefits of functional foods to humans have been explored (Muhammad et al., 2018). Among the several important beneficial effects of functional foods include their potential to prevent and treat COVID-19. Here, we present functional foods that have been shown to modulate ACE2 (Table 2).

**TABLE 2:** Functional foods known to modulate ACE2.

Functional food	Bioactive compound	Method of assessment	References
Fatty acids	Omega- FA 3	*In vivo*	(Gupte, 2011; Ulu et al., 2013)
Food-derived peptides			
Soybeans egg-white *Spirulina platensis* (blue-green algae)	Nicotianamine peptide LY peptide RALP peptide GHS peptide IRW peptide IQP peptide VEP	*In vitro*, *in vivo*	(Majumder et al., 2015; Takahashi et al., 2015; Zheng et al., 2017; He et al., 2019; Liao, 2019; Liao et al., 2019)

GHS, Gly His-Ser; IQP, Ile-Gln-Pro; IRW, Ile-Arg-Trp; LY, Leu-Tyr; RALP, Arg-Ala-Leu-Pro; VEP, Val-Glu-Pro.

### Fatty Acids (FA)

Naturally occurring fatty acids have been previously reported to have health benefits in several disease states. Omega-3 FA, fixed oils, oils obtained from nuts, coconut, soy, sesame, and fruits are common sources of naturally occurring FA. Omega-3 FA are a class of polyunsaturated fatty acids (PUFA) that are known to have beneficial effects in the prevention and management of various diseases (Muhammad et al., 2018). The two major biologically active types of omega-3 FA are eicosapentaenoic acid and docosahexaenoic acid (DHA) (Daak et al., 2020). The ACE2 activities of these bioactive agents have been demonstrated in previous studies. Ulu and co-workers have shown that diet rich in omega-3 FA attenuated inflammation in angiotensin-II dependent hypertension by up-regulating the ACE2 activity (Ulu et al., 2013). In another study, Gupte. (2011) revealed that omega-3 FA upregulated the expression of ACE2 mRNA in adipocytes (Gupte, 2011). As we shall discuss, following the emergence of COVID-19, several essential oils from functional foods have also been identified as ACE2 modulators (Senthil Kumar et al., 2020; Thuy et al., 2020).

### Food-Derived Peptides

Previous indications suggest food-derived bioactive peptides can regulate the body’s physiological functions. These food-derived peptides have been demonstrated to exert potential health benefits to humans and thus serve as functional foods. In relation to this, soy proteins isolated from soybeans (*Glycine max* L.) were demonstrated to have ACE2 inhibitory properties (Table 2). In the first ACE2 inhibitory study using food products, Takahashi et al. (2015) demonstrated the ACE2 inhibitory activity of soybean (*Glycine max* L.) and its isolated bioactive protein. In their study, the bioactive protein, nicotianamine, intensely inhibited the ACE2 activity at 84 nM concentration. Similarly, bioactive peptides from rapeseed, Leu-Tyr (LY), Arg-Ala-Leu-Pro (RALP) and Gly His-Ser (GHS), have been shown to modulate the ACE2 activity in spontaneously hypertensive rats (He et al., 2019). In this study, ACE2 expression markedly increased following oral administration of the peptides (30 mg/kg) both at the gene and protein levels. A related study had also demonstrated that administration of bioactive peptides (10 mg/kg/day) from blue-green algae, *Spirulina platensis (Arthrospira platensis)*, upregulates the expression of ACE2 in spontaneously hypertensive rats (Zheng et al., 2017). The fact that these specific peptides modulate ACE2 expression and subsequent activity hints to their bioactivity. However, the pharmacokinetics and mechanisms behind these physiological effects remain largely unknown and may require extensive investigations before becoming practically applicable. Additionally, food components interact with peptides in different ways that may affect the availability of peptides within the matrix (Chakrabarti et al., 2018). As such, to recommend foods rich in ACE2 modulatory peptides, further research is needed to determine the release, stability and mechanisms of bioactive peptides following normal digestion. Furthermore, hydrolysates from pre-digestion when subjected to normal digestion may produce different peptide sets (Chakrabarti et al., 2018). In this case, investigations are needed to demonstrate if the identified physiological effects are abolished, unabolished or changed.

Furthermore, evidences have indicated that the egg-white-derived antihypertensive peptide Ile-Arg-Trp induces the expression of ACE2 and decreases proinflammatory gene expression in mesenteric arteries of spontaneously hypertensive rats (Majumder et al., 2015). A similar *in vivo* study had further demonstrated that the tripeptide IRW elicits vasorelaxation and abolishes vascular inflammation by increasing the levels of circulatory ACE2 and Ang (1–7), potentiating its protective activity. (Liao et al., 2019). Notably, IRW is less-susceptible to peptide modifications by digestive enzymes (Bejjani and Wu, 2013). Findings from these studies demonstrate the previously established beneficial effects of food-derived proteins and other components in modulating ACE2 activity that subsequently influences the body physiology in a way that could antagonize the development of COVID-19. Though these peptides for intake may be largely acceptable due to natural source, a minimal tissue bioavailability of most peptides limits theirs use as therapeutic agents (Campos et al., 2011). To address this, approaches like microencapsulation, use of enzyme inhibitors, may be needed to achieve effective oral delivery. Further *in vivo* studies are also needed to understand the immune response to peptides and human trials may become necessary to validate efficacy. However, the production of such synthetic bioactive peptides faces limitations in expense and requires newer low-cost innovations.

### Phytomedicinal Plants, COVID-19 and the Effects on ACE2

**FIGURE 4:**
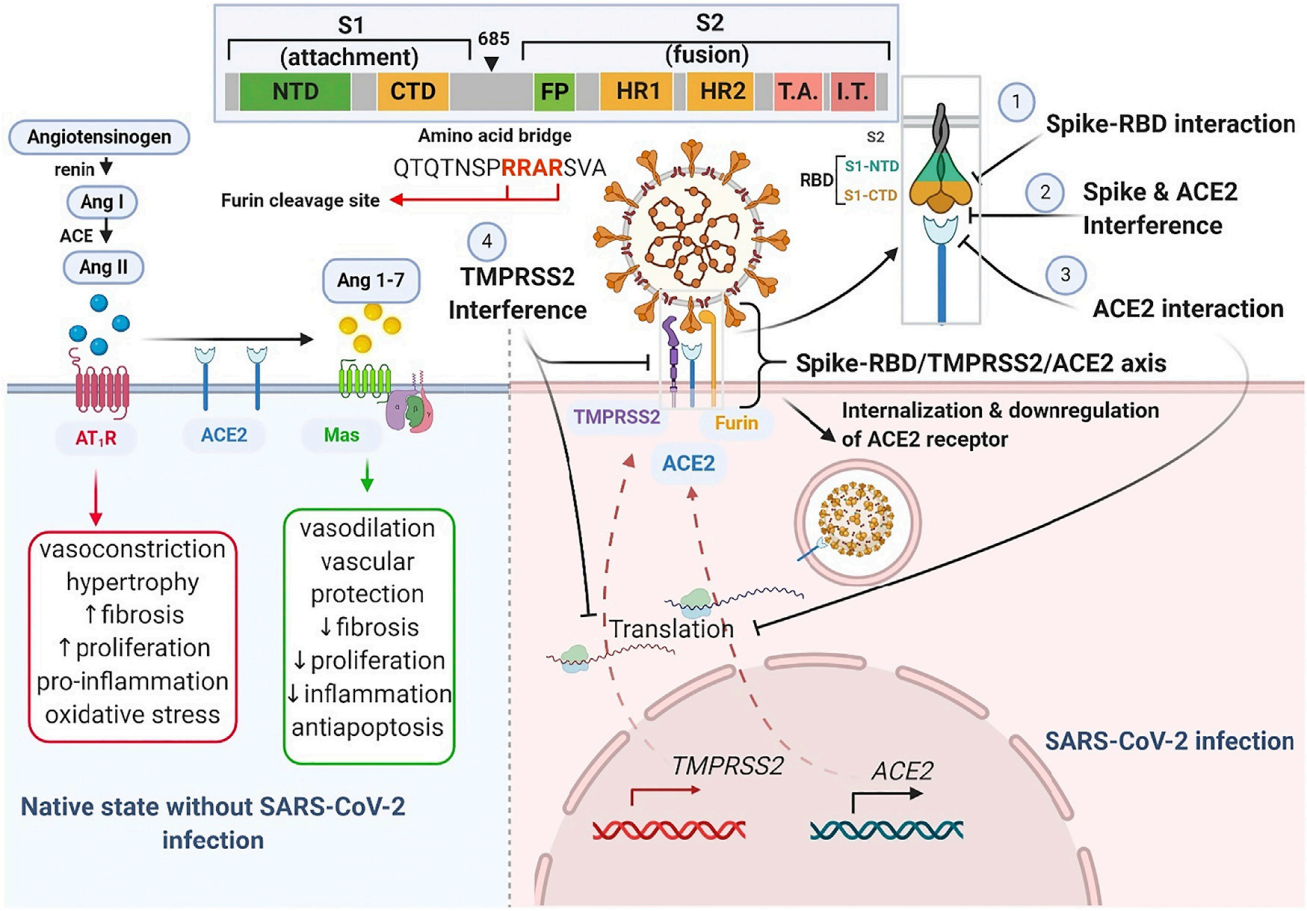
Schematic drawing of Spike-RBD/TMPRSS2/ACE2 axis. The left half illustrates the native counter-interaction between the RAS pathway and ACE2. The right half highlights four targets of phytomedicine against the Spike-RBD/TMPRSS2/ACE2 axis following SARS-CoV-2 infection. The inset shows the furin cleavage site (-RRAR-) of a spike glycoprotein.

At this time, several claims have been made on herbal-based traditional medicines or phytomedicines as remedies to COVID-19. These herbs and products include COVID organics (Madagascar), claims on *Andrographis paniculata* (Burm.f.) Nees, *Tinospora crispa* (L.) Hook. f*.* and Thomson*,* and *Gymnanthemum amygdalinum* (Delile) Sch. Bip (DR-Congo), turmeric (*Curcuma longa* L.), a Sri Lankan herbal drink, and some Tanzanian claims about recipes from lemon grass, ginger, and neem leaves. Notably, like synthetic agents for COVID-19 such as remdesivir, dexamethasone and chloroquine, some of these herbs and products are recently being supported by preliminary evidence and have been suggested for further empirical assessments (Borkotoky and Banerjee, 2020; Enmozhi et al., 2020; Rakib et al., 2020). With a focus on identifying and supporting the development of plant products having some level of scientific evidence in the context of ACE2-centered SARS-CoV-2 fusion-entry, we describe three classes of phytomedicinal agents, based on the target used to modulate viral tropism (virion fusion and entry) along the Spike-RBD/TMPRSS2/ACE2 axis. These phytomedicinal agents include those that may 1) interfere with host cell surface proteins (ACE-2 receptor and TMPRSS2 protease), 2) interface with viral spike glycoprotein, or 3) interrupt spike-RBD/ACE2 interaction **(** Figure 4 **).**

### The Spike-RBD/TMPRSS2/ACE2 Axis

**FIGURE 5:**
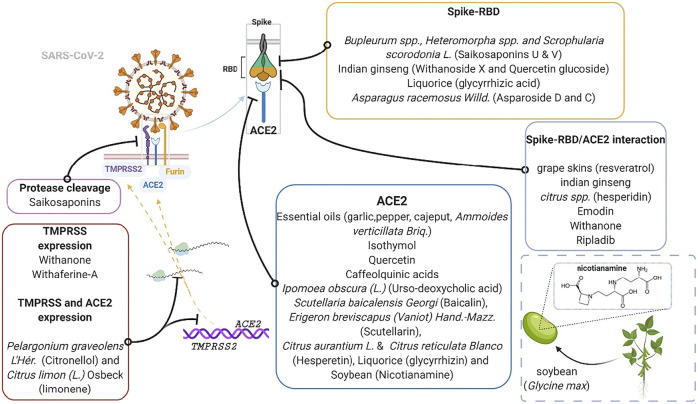
Plants and plant products that modulate entry of SARS-CoV-2. The inset shows nicotianamine derived from soy, a product of soybean (*Glycine* max L.). ACE2, Angiotensin converting enzyme two; RBD, receptor binding domain; TMPRSS2, transmembrane protease serine 2. Phytomedicinal targets of the Spike-RBD/TMPRSS2/ACE2 axis.

The SARS-CoV-2 fusion and entry, which is characteristic of COVID-19, marks a phytomedicinal target for COVID-19 therapy. Following SARS-CoV-2 spike-RBD attachment to the host cell ACE2 receptor, the membrane bound activator protease, TMPRSS2, cleaves the viral spike protein and facilitates its fusion with the host cell membrane (Hoffmann et al., 2020). This constitutes the Spike-RBD/TMPRSS2/ACE2 axis and the cleavage (or priming) that occurs at the junction between subunit one and two of the spike protein precedes internalization. The Spike-RBD/TMPRSS2/ACE2 axis presents several interventional targets for natural products at the 1) receptor binding motif (RBM) of the receptor binding domain (RBD) of the spike glycoprotein, 2) ACE2 receptor active site that recognizes the RBD and 4) TMPRSS2 protease (Figure 5)**.**

### Interference With ACE2 Activity–ACE2

Several plant products have shown potential in interfering with the activity of ACE2 (Chen and Du, 2020; Joshi et al., 2020; Poochi et al., 2020). For example, the essential oils from *Allium sativum L* (garlic), *Ammoides pusilla (Brot.) Breistr.*, *Melaleuca cajuputi Maton* and *Sm. ex R. Powell* (cajeput) and *Piper* spp (pepper) like *Piper nigrum L.* and *Piper retrofractum Vahl,* can potentially latch onto ACE2 and competitively inhibit viral binding and entry (Abdelli et al., 2020; Gutierrez-Villagomez et al., 2020; My et al., 2020; Thuy et al., 2020). These inhibitory effects have been attributed to the presence of certain ligands (Table 3). For instance, Senthil Kumar et al. (2020) demonstrated that essential oils from geranium and lemon prove to be effective against ACE2 by downregulating its mRNA and protein expressions in epithelial cells. Further gas chromatography mass spectrometry (GC-MS) analysis identified citronellol, geraniol and neryl acetate in geranium oils, and limonene in lemon oils as the major ligands (particularly citronellol and limonene) responsible for the downregulation of ACE2 expression in HT-29 cells. Same ligands were also identified to inhibit TMPRSS2 (Senthil Kumar et al., 2020). Notably, not all ligands that bind with ACE2 inhibit its enzymatic activity (Williamson and Kerimi, 2020). The fact that some of these essential oils bear several molecules with binding capacity hints to some possible synergistic effect that may account for the overall efficacy of essential oils. Interestingly, some of these interactive ligands can be synthesized (Gutekunst et al., 2012; Liu et al., 2012), indicating a vastly unexplored potential. Additionally, in a novel attempt, Rattanapisit and co-workers used a plant expression system (*Nicotiana benthamiana* Domin) to develop and characterize the rapid production of recombinant RBD as a biopharmaceutical capable of neutralizing viral entry by specifically binding to ACE2 (Rattanapisit et al., 2020). Such use of plants as platforms for recombinant protein production, offers benefit over animal-based platforms in terms of cost and is also advantaged in its flexibility, rapid scalability and safety. The recombinant RBD possibly exhibit *in vivo* neutralization activity similar to recently developed capsid-like particle-based vaccines against SARS-CoV-2 invasion (Fougeroux et al., 2021), and may represent another turning point for COVID-19 therapeutics.

**TABLE 3:** Plant derivatives with potential effect on the Spike-RBD/TMPRSS2/ACE2 axis.

Target	Plant (bioactive phytoconstituent/product)	Study type	Efficacious dose(s)	Biological test	Mechanistic effect	Findings	References	Comment
Interfere with ACE2 activity	*Nicotiana benthamiana* domin (recombinant RBD)	*In silico and In vitro* (vero E6 cells)	NA	Recombinant mAb RBD production in a plant expression system. Neutralization efficiency against positive sera	Binds to ACE2	Specific binding to the SARS-CoV-2 receptor and its neutralization	Rattanapisit et al. (2020)	Suggested consideration of this plant derived recombinant RBD for the development of vaccines and viral detection/diagnostic reagents
	*Pelargonium graveolens* L'Hér. (Citronellol) and *Citrus × limon* (L.) osbeck (Limonene)^a^	*In vitro* (HT-29 cell line)	50 μg/ml geranium oil and 25 μg/ml lemon oil	Gene expression profile (mRNA and protein)	Downregulates the expression of ACE2 and TMPRSS2	Significant inhibition of ACE2 and TMPRSS2 in epithelial cells to protect against SARS-CoV-2 invasion	Senthil Kumar et al. (2020)	Citronellol and limonene were the most potent of eight ACE2 inhibitory oil extracts and had dose dependent effects
	*Momordica dioica* roxb. ex willd (catechin, quercetin, hederagenin and oleanolic acid)	*In silico and In vitro*	NA	Molecular docking and *in silico* ADME predictions methods	Docking to ACE2	The constituent bioactive flavonoids (catechin and quercetin) and triterpenoids (hederagenin and oleanolic acid) inhibit ACE2 and DPP4 receptors	Sakshi et al. (2021)	Constituent flavonoids have better affinities than standard remdesivir, favipiravir and hydroxychloroquine
	*Valeriana jatamansi* jones ex roxb. (Hesperidin), *Oroxylum indicum* (L.) kurz (chrysin), *Rheum australe* D.Don (emodin)	*In silico*	NA	Molecular docking and molecular dynamics	Allosterically binds to ACE2 and can also destabilize spike-ACE2 interaction	Ligands (especially hesperidin) triggers conformational changes that causes spike-ACE2 fragment to be unstable	Basu et al. (2020)	Spike inhibitory capacity similar to that of docked chloroquine and hydroxychloroquine
	*Artemisia absinthium* L. (anabsinthin, absinthin, dicaffeoylquinic acids), *Syzygium aromaticum* (L.) merr. and L.M.Perry (3-0-caffeoylquinic), *Phaseolus vulgaris* L. (quercetin 3-glucuronide-7-glucoside, quercetin 3-vicianoside, isosakuranetin 7-O-neohesperidoside) and *Inula helenium* L. (Quercetin-7-O-galactoside, 3,5-dicaffeoylquinic acid, 3,4,5-tricaffeoylquinic acid)	*In silico*	NA	Molecular modeling/docking and dynamic simulations	High affinity binding to pocket of the active site of ACE2	Ligands could inhibit viral fusion	Joshi et al. (2020)	Compounds demonstrated good intestinal and brain permeability. Also showed no carcinogenic tendency
	*Allium sativum* L. (diallyl tetrasulfide and trisulfide, 2-propenyl propyl)^a^	*In silico*	N/A	Molecular modeling/docking	Binds to ACE2 receptor	Could inhibit viral entry and infectivity	Thuy et al. (2020)	Ligands are the two most potent of seventeen inhibitors of ACE2 gotten from essential oil of plant
	*Piper* sp*.* (pipercyclobutanamide B, a and nigramide Q) like *Piper nigrum* L. and *Piper retrofractum* vahl	*In silico*	NA	Molecular modeling/docking and dynamic simulations	Docks closely to active site of ACE2	These dimeric piperamides of essential oil could possibly inhibit ACE2 mediated entry of SARS CoV2	Gutierrez-Villagomez et al. (2020)	Exhibit potential drug likeness based on ADME. Pipercyclobutanamide B (most potent) docked along duct to ACE2 active site
	*Ipomoea obscura* (L.) ker gawl. (Urso-deoxycholic acid)^a^	*In silico*	N/A	Molecular modeling/docking	Bind to ACE2 receptor	Could inhibit viral entry and infectivity	Poochi et al. (2020)	This is the most potent of five possibly bioactive ACE2 inhibitors from ethanolic extract of the plant
	*Ammoides verticillata* (desf.) briq. -From Algeria (isothymol)	*In silico*	NA	Molecular modeling/docking	Binds to ACE2 receptor	High affinity and can inhibit ACE2 better than captopril and chloroquine drugs	Abdelli et al. (2020)	Suggest oil is one of the richest natural sources of isothymol. Good ADMET
	*Melaleuca cajuputi* maton & sm. ex R. Powell (terpineol, guaiol and linalool)^a^	*In silico*	NA	Molecular modeling/docking and dynamic simulations	Latch to the active site of ACE2	Lots of convergence points and the inhibitory intensity of these compounds on ACE2 could prevent viral invasion	My et al. (2020)	Out of ten inhibitory substances, these three have the most potent effect on ACE2. Guaiol is also present in guaiacum and cypress pine oils
	*Scutellaria baicalensis* georgi (baicalin), *Erigeron breviscapus* (vaniot) hand-mazz. (Scutellarin), *Citrus × aurantium* L. *and Citrus reticulata* blanco (hesperetin), liquorice; *Glycyrrhiza uralensis* fisch. ex DC. (glycyrrhizin) and soybean; *Glycine* max (L.) merr. (Nicotianamine)	*In silico*	NA	Molecular modeling/docking and dynamic simulations	Latch to the active site of ACE2	Potential to bind ACE2 and hinder viral entry	Chen and Du (2020)	Nicotianamine is “soybean ACE2 inhibitor” (ACE2iSB)
	Gancao*: Glycyrrhiza* spp. and chaihu*: Bupleurum* spp. (glyasperin F and isorhamnetin)	*In silico*	NA	Molecular docking	Bind to ACE2	Latch onto site 1 and site 2 of ACE2	Ren et al. (2020)	Suggest the ligands, glyasperin F and isorhamnetin, account for strong binding affinity
Interface with the viral spike glycoprotein and its RBD	Liquorice; *Glycyrrhiza glabra* L*.* (glycyrrhizic acid)	*In silico*	NA	Molecular modeling/docking and dynamic simulations	Bind to cavity of prefusion spike glycoprotein	High binding affinity to spike protein may block viral fusion to ACE2	Sinha et al. (2020a)	High protein-ligand stability. Most potent of six interactive ligands
	*Bupleurum* spp.*, Heteromorpha* spp. *and Scrophularia scorodonia* L*.* (saikosaponins U and V)^a^	*In silico*	NA	Molecular modeling/docking and dynamic simulations	Binds to RBD and cleavage site of the spike glycoprotein	May inhibit viral entry by interfering with virion-receptor binding and protease cleavage	Sinha et al. (2020b)	Out of 23 saikosaponins, had the most potent latching affinity to active site of spike glycoprotein (i.e. RBD)
	Indian ginseng: *Withania somnifera* (L.) dunal (withanoside X and quercetin glucoside)^a^	*In silico*	NA	Molecular modeling/docking and dynamic simulations	Binds receptor binding domain of prefusion spike protein from SARS-CoV-2	Favourable interaction with receptor binding motif (RBM) of RBD to block viral fusion	Chikhale et al. (2020a)	Ligands were potent (out of 17) inhibitors of SARS-CoV-2 spike glycoprotein
	*Asparagus racemosus* willd. (Asparoside D and C)	*In silico*	NA	Molecular modeling/docking and dynamic simulations	Bind to spike RBD	Good affinity and stable docking of spike RBD	Chikhale et al. (2020b)	Higher binding affinity than remdesivir (standard drug)
Interrupting the spike-rbd/ace2 interaction	*Withania somnifera* (L.) dunal (withanone)	*In silico*	NA	Molecular modeling/docking	Interrupts at the junction between ACE2 receptor and viral S-RBD	Decreased binding free energies, destabilized salt bridges, hence blocks and weaken SARS CoV2 entry and infectivity	Balkrishna et al. (2020)	Suggest plant as first choice herb in curbing COVID-19
	*Diplocyclos palmatus* (L.) leaf extract (ripladib)	*In silico*	NA	Molecular modeling/docking	Interrupts at the RBD of Spike-ACE2 complex	Predicted strong binding at RBD interface to block Spike-ACE2 interactions and viral entry	Alexpandi et al. (2020)	ADMET analysis indicate good pharmacokinetic properties
	*Citrus* spp. (hesperidin)	*In silico*	NA	Molecular modeling/docking	Binds to both RBD and ACE2 receptor	The flavonoid can possibly interrupt RBD/ACE2 interface to abrogate entry	Utomo et al. (2020)	The peel of Citrus sp. represents the most abundant methoxy flavonoid (hesperidin) store of the plant
	Grape skin: *Vitis vinifera* L. (resveratrol)	*In silico*	NA	Molecular modeling/docking and dynamic simulations	Bind tightly and interfere with viral S protein/ACE2 receptor complex	Highly stable binding and selectivity to viral protein/ACE2 receptor complex. Disrupts the spike protein	Wahedi et al. (2020)	Most potent of four stilbene-based natural compounds
Interfere with the host membrane protease	*Aframomum melegueta* K.Schum. (quercetin, apigenin)	*In silico and In vitro*	30, 10, 3, 1, 0.3 mg/L. From fruit (with seed)	Docking. *In vitro* inhibition of recombinant soluble human furin. Immuno-blotting	Disrupts spike glycoprotein/receptor interaction by inhibiting furin cleavage	Metabolites inhibited furin dependent pre-glycoprotein processing by possibly blocking furin recognition site	Omotuyi et al. (2020)	Suggested bioactive influence of flavonoids (like quercetin, apigenin). Good ADMET sores
	*Withania somnifera* (L.) dunal (withanone and Withaferine-A)	*In silico*	NA	Molecular modeling/docking	Binds to TMPRSS2 catalytic site (Wi-N > Wi-A), alters allosteric site and also downregulates TMPRSS2 transcription	Predicted multiple action in blocking SARS CoV2 cell entry and propagation by inhibiting TMPRSS2	Kumar et al. (2020c)	Possible drug-able agents for prevention and therapeutics

a These compounds were found to be the most potent of several others.

**Abbreviations:** ACE2, Angiotensin converting enzyme two; ADME/T, Absorption, Distribution, Metabolism, Excretion/and Toxicity; DPP4, Dipeptidyl peptidase four; mAb, monoclonal antibody; NA, not available; PK, Pharmacokinetic; S-RBD, Spike glycoprotein receptor binding domain; TMPRSS2, Transmembrane protease serine two; Wi-A, Withaferine-A; Wi-N, Withanone.

### Interference With ACE-2 Activity - ACE2 and Angiotensin

ACE2 is known to counter the vasoconstrictive and other detrimental effects of the Angiotensin-1/ACE/Angiotensin-II/AT-1 Receptor axis which is a component of the RAS. ACE2 converts angiotensin-1 and -II into angiotensin one to seven, which acts on Mas receptors. This shunt represents the renin angiotensin system suppressive axis called “ACE2/Ang 1–7/Mas receptor axis,” which produces vasodilatory and other beneficial effects that represent the protective effect of ACE2 on the body (Furuhashi et al., 2020). As stated earlier, the fusion of SARS-CoV-2 to ACE2 and its subsequent entry downregulates the levels of ACE2 thereby potentiating the detrimental activity of RAS. This makes the RAS an important marker to look at when testing possible COVID-19 therapies. Currently, a Tomeka® Prevention Trial (TPT) is underway in DR Congo to assess the effect of using Tomeka® regimen for COVID-19 on markers of the RAS such as angiotensin-II and -(1–7) (ClinicalTrials.gov identifier: NCT04537585). Tomeka® is an herbal mixture made from soy (from soybean; *Glycine* max L.), sorghum (*Sorghum bicolor* L.), maze (*Zea mays* L.) and mushrooms (*Agaricus bisporus* L.). Nicotianamine had been highlighted as the bioactive phytoconstituent of soy responsible for its potent inhibition of ACE2 (Figure 5) and it was hence, called “soybean ACE2 inhibitor - ACE2iSB” (Chen and Du, 2020).

### Interface With the Viral Spike Glycoprotein and Its RBD

For COVID-19, it is more logical to find phytomedicinal agents that specifically target the interaction of RBD at the Spike/ACE2 complex and not particular to ACE2. This caution is because agents solely targeting ACE2 may interfere with ACE2 enzyme activity and its complex protective functions (Kai and Kai, 2020). Natural products with significant RBD binding affinity compared to ACE2 can dominate over ACE2 in latching onto RBD, thereby blocking viral attachment, fusion and entry.

Preliminary data based on molecular docking analyses predict several natural plant products as RBD interface binding compounds (Chikhale et al., 2020a; Chikhale et al., 2020b; Sinha et al., 2020a; Sinha et al., 2020b; Table 3). Most data come from “conceivable therapeutics” through *in silico* molecular analysis of available structural macromolecules. An advantage to the preliminary *in silico* screening of substances is that pharmacokinetic properties, such as safety could be easily established, shifting priority to the *in vitro* confirmation of efficacy. Thus, preliminary evidences necessitate further *in vitro* and *in vivo* investigations. Notably, Wang and co-workers have recently identified AXL, a transmembrane tyrosine-protein kinase, as a supportive pulmonary entry receptor for the SARS-CoV-2 which may also interact with the spike glycoprotein, but at its NTD region (Wang et al., 2021). This represents another potential target for plant based therapeutic investigations.

### Interrupting the Spike-RBD/ACE2 Interaction

Studies suggest that 1) resveratrol from grape skins, 2) ripladib from the leaf extracts of the native bryoni, and 3) withanone from Indian ginseng, can selectively bind and inhibit both RBD and ACE2 (Alexpandi et al., 2020; Balkrishna et al., 2020; Wahedi et al., 2020). These ligands can potentially inhibit the Spike-RBD/TMPRSS2/ACE2 axis simultaneously at the RBD and ACE2. Moreover, multiple ligand activity on the said axis indicates a higher likelihood of getting some potent alternative for COVID-19. Ho et al. (2007), in an *in vitro* study, established that emodin, from *Rheum* sp*.* and *Polygonium* sp*.* abolish the interaction between 2002/2003 SARS-CoV spike protein and ACE2 in a dose-dependent manner. Given the level of homology between the two coronaviruses, an investigation into the influence of emodin against the interaction of the SARS-CoV-2 spike and ACE2 is necessary.

Notably, the components of some plants may differ based on the plant part used (Kumar et al., 2020a). For instance, the *Citrus* spp. peel represents the most abundant store of hesperidin, another possibly potent inhibitor of the RBD and ACE2 interaction (Utomo et al., 2020). Consequently, the hesperidin ligand is currently scheduled for a randomized double-blind controlled trial in Egypt (ClinicalTrials.gov identifier: NCT04452799) (Haggag et al., 2020; Okasha, 2020). Knowledge of such should guide research and may afford some better protection against SARS-CoV-2 invasion.

### Interfering With the Host Membrane Enzymes; TMPRSS2, Furin and ADAM-17

The protease cleavage site between the S1/S2 subunits of the spike protein bears an arginine-arginine-alanine-arginine (RRAR) sequence (Wang et al., 2020b) (as shown in Figure 4)**.** What is now known is that furin, a proprotein convertase enzyme, performs cumulative role with TMPRSS2 by pre-activating this site, aiding viral entry (Bestle et al., 2020; Shang et al., 2020). One molecular simulation study suggests that saikosaponins interact significantly with the arginine residues of the cleavage site to prevent S1/S2 protein cleavage, hindering the virion-cell fusion and viral entry (Sinha et al., 2020b). Indeed, the furin pre-activation of spike protein facilitates viral entry particularly in cells with low levels of expressed TMPRSS2 (Lippi et al., 2020; Shang et al., 2020). Omotuyi et al. (2020) demonstrated in a conformational and *in vitro* study, that processed fruit (with seed) from *Aframomum melegueta* K.Schum., an African resource, and its secondary metabolites mitigates SARS-CoV-2 entry by inhibiting furin. Indeed, some proteinaceous plant products (peptides) and metabolites (flavonoids such as rutin, naringin, methylhesperidin and baikalin), have exhibited anti-protease potencies and have been implicated for disease treatment (Majumdar et al., 2010; Hellinger and Gruber, 2019). In addition, ADAM-17, a membrane metallopeptidase opposes viral entry by cleaving off the extracellular domain of ACE2 receptor. The ACE2 domain that is shed off retains some enzyme activity and can convert angiotensin II to angiotensin 1–7 (Palau et al., 2020; Williamson and Kerimi, 2020). Hence, a closer look into the ability of plant-based ligands to upregulate ADAM-17 represents a potential angle for exploration, which may achieve satisfactory outcomes.

## Safety

Phytomedicinal agents investigated against the Spike-RBD/TMPRSS2/ACE2 axis have displayed beneficial pharmacokinetic properties when subjected to *in silico* analysis of Absorption, Distribution, Metabolism, Excretion and Toxicity (ADMET) (Abdelli et al., 2020; Alexpandi et al., 2020; Gutierrez-Villagomez et al., 2020). These preliminary findings hint to their safety and drug-like potential. However, in view of the limitation of virtual screening, further *in vitro and in vivo* experiments are needed to verify results and provide experimental basis for the research and development/promotion of natural antiviral drugs/nutrition. The cytotoxic status of a plant is also an important safety concern. An *in vitro* study showed that geranium and lemon essential oils inhibited the expression of ACE2 and TMPRSS2 at mRNA and protein levels with no cytotoxic effects on human cell (Senthil Kumar et al., 2020). Additionally, most plant products highlighted are very promising agents keeping in view their pre-reported biological benefits. Thus, further experiments are warranted based on previous research findings and the reported biological safety of these natural compounds. Moreover, these natural compounds may be synthesized and made readily available for immediate testing.

### Formalizing the Use of COVID-19 Phytomedicines

Following the wide use of phytomedicines as alternatives for preventing and treating COVID-19 in Africa, the Africa Center for disease Control and Prevention (Africa CDC) recommended the development of state-based herbal registries. The mobilization of funds, technical research support and proper communication for the development of such proclaimed remedies have also been emphasized (Africa CDC, 2020). These activities can help guide the establishment of botanical assets in the management of COVID-19.

Similar to what was introduced during the 2002/2003 SARS-COV outbreak, a Chinese traditional medicine program challenged its experts with the development of preventive and treatment strategies for COVID-19. A similar global effort to introduce scientifically backed herbal remedies to combat COVID-19 alongside conventional treatment will prove helpful. Though these herbals are promising options in decreasing virion entry and the course of infection, caution must be established and tests must be conducted to avoid toxic and non-efficacious regimens. Undeniably, clinically proven therapeutics remain the gold standard for treatment. As such, efforts to engage preliminary and pre-clinically supported herbs of minimal side effects and cost with randomized controlled trials should be considered.

## Conclusions and Future Perspectives

Natural products are of prominence and have been adopted for traditional use across many African and Asian countries. Aside policies and a bundle of other factors, the relatively lesser COVID-19 burden observed in regions of Africa and Asia could be influenced by certain potentiators of self-care such as cost of medication, interest, beliefs, dissatisfaction in synthetic medications and taking responsibility of one’s health, which push toward ethnobotanical use for COVID-19; a scenario where “the kitchen cabinet becomes thy medicine cabinet.” Of concern, the undeniably increasing demand for plant products is occurring globally at a time threatened by plant extinctions; particularly of wild plants due to excess harvest, habitat degradation, specie invasion, low growth, low abundance, and/or susceptibility to disease. This coupled with the fact that at least one potential drug candidate is lost biennially emphasizes the need for conservation and resource management strategies (Chen et al., 2016). This is most possible given the current state of biotechnology and must be considered particularly in developing countries where about 80% of the populace adopt herbal products for basic healthcare (Moyo et al., 2015; Chen et al., 2016). The overall indicators show that a number of plants and their products possess multimodal influence on viral tropism. Beside the use of plant decoctions, some phytoconstituents previously highlighted, indicate potentially mitigating effects against viral entry by directly or indirectly modulating the ACE2 activity at the Spike-RBD/TMPRSS2/ACE2 axis. This perspective has implications for traditional medicine (TM) and we believe will support and inform ongoing and upcoming investigations on the management of COVID-19. More so, some of the highlighted ligands have been tested in the past and documented to be beneficial across comorbidities which may accompany COVID-19. Perhaps, this lightens the need for further studies before clinical trials. Other benefits including availability, affordability and safety, support the development of these phytomedicinal therapies; a step toward attaining the third united nations sustainable developmental goal - “good health and wellbeing for all.”

## Abbreviations

ACE2, Angiotensin converting enzyme two; ADME/T, Absorption, Distribution, Metabolism, Excretion/and Toxicity; DPP4, Dipeptidyl peptidase four; mAb, monoclonal antibody; NA, not available; PK, Pharmacokinetic; S-RBD, Spike glycoprotein receptor binding domain; TMPRSS2, Transmembrane protease serine two; Wi-A, Withaferine-A; Wi-N, Withanone.

## References

[B1] Abdelli I. Hassani F. Bekkel Brikci S. Ghalem S. (2020). In silico study the inhibition of angiotensin converting enzyme 2 receptor of COVID-19 by Ammoides verticillata components harvested from Western Algeria. J. Biomol. Struct. Dyn., 1–14. 10.1080/07391102.2020.1763199 PMC7232889 32362217

[B2] Africa C. D. C. (2020). Statement on herbal remedies and medicines for prevention and treatment of COVID-19. Africa centres for disease control and prevention: african union Available from: https://africacdc.org/download/statement-on-herbal-remedies-and-medicines-for-prevention-and-treatment-of-covid-19-2/ (Accessed October 11, 2020).

[B3] Alexpandi R. De Mesquita J. F. Pandian S. K. Ravi A. V. (2020). Quinolines-based SARS-CoV-2 3CLpro and RdRp inhibitors and spike-RBD-ACE2 inhibitor for drug-repurposing against COVID-19: an in silico analysis. Front. Microbiol. 11. 10.3389/fmicb.2020.01796 PMC7390959 32793181

[B4] Balkrishna A. Pokhrel S. Singh J. Varshney A. (2020). Withanone from Withania somnifera may inhibit novel Coronavirus (COVID-19) entry by disrupting interactions between viral S-protein receptor binding domain and host ACE2 receptor. Res. Square . 10.21203/rs.3.rs-17806/v1

[B5] Basu A. Sarkar A. Maulik U. (2020). Molecular docking study of potential phytochemicals and their effects on the complex of SARS-CoV2 spike protein and human ACE2. Sci. Rep. 10 (1), 17699. 10.1038/s41598-020-74715-4 33077836 PMC7573581

[B6] Bejjani S. Wu J. (2013). Transport of IRW, an ovotransferrin-derived antihypertensive peptide, in human intestinal epithelial caco-2 cells. J. Agric. Food Chem. 61 (7), 1487–1492. 10.1021/jf302904t 23298184

[B7] Bestle D. Heindl M. R. Limburg H. Van Lam van T. Pilgram O. Moulton H. (2020). TMPRSS2 and furin are both essential for proteolytic activation of SARS-CoV-2 in human airway cells. Life Sci. alliance 3 (9). 10.26508/lsa.202000786 PMC7383062 32703818

[B8] Bhalla V. Blish C. A. South A. M. (2020). A historical perspective on ACE2 in the COVID-19 era. J. Hum. Hypertens. 10.1038/s41371-020-00459-3 PMC7735396 33318644

[B9] Borkotoky S. Banerjee M. (2020). A computational prediction of SARS-CoV-2 structural protein inhibitors from Azadirachta indica (Neem). J. Biomol. Struct. Dyn. 1-17 10.1080/07391102.2020.1774419 PMC7311162 32462988

[B10] Bourgonje A. R. Abdulle A. E. Timens W. Hillebrands J. L. Navis G. J. Gordijn S. J. (2020). Angiotensin‐converting enzyme 2 ( ACE2 ), SARS‐CoV ‐2 and the pathophysiology of coronavirus disease 2019 ( COVID ‐19). J. Pathol. 251 (3), 228–248. 10.1002/path.5471 32418199 PMC7276767

[B11] Campos M. Guerrero L. Betancur D. Hernandez-Escalante V. (2011). Bioavailability of bioactive peptides. Food Rev. Int. 27, 213–226. 10.1080/87559129.2011.563395

[B13] Chakrabarti S. Guha S. Majumder K. (2018). Food-derived bioactive peptides in human health: challenges and opportunities. Nutrients 10 (11), 1738. 10.3390/nu10111738 PMC6265732 30424533

[B14] Chen H. Du Q. (2020). Potential natural compounds for preventing SARS-CoV-2 (2019-nCoV) infection. 10.20944/preprints202001.0358.v3

[B16] Chen L. Li X. Chen M. Feng Y. Xiong C. (2020a). The ACE2 expression in human heart indicates new potential mechanism of heart injury among patients infected with SARS-CoV-2. Cardiovasc. Res. 116 (6), 1097–1100. 10.1093/cvr/cvaa078 32227090 PMC7184507

[B17] Chen S. L. Yu H. Luo H. M. Wu Q. Li C. F. Steinmetz A. (2016). Conservation and sustainable use of medicinal plants: problems, progress, and prospects. Chin. Med. 11, 37. 10.1186/s13020-016-0108-7 27478496 PMC4967523

[B18] Chen Y. Guo Y. Pan Y. Zhao Z. J. (2020b). Structure analysis of the receptor binding of 2019-nCoV. Biochem. Biophysical Res. Commun. 525 (1), 135–140. 10.1016/j.bbrc.2020.02.071 PMC7092824 32081428

[B19] Chen Y. Liu Q. Guo D. (2020c). Emerging coronaviruses: genome structure, replication, and pathogenesis. J. Med. Virol. 92 (4), 418–423. 10.1002/jmv.25681 31967327 PMC7167049

[B20] Cheng L. Zheng W. Li M. Huang J. Bao S. Xu Q. (2020). Citrus fruits are rich in flavonoids for immunoregulation and potential targeting ACE2. Preprints 10.1007/s13659-022-00325-4 PMC8844329 35157175

[B21] Chikhale R. V. Gurav S. S. Patil R. B. Sinha S. K. Prasad S. K. Shakya A. (2020a). Sars-cov-2 host entry and replication inhibitors from Indian ginseng: an in-silico approach. J. Biomol. Struct. Dyn., 1–12 . 10.1080/07391102.2020.1778539 PMC7332873 32568012

[B22] ChikhaleR. V.SinhaS. K.PatilR. B.PrasadS. K.ShakyaA.GuravN. (2020b). In-silico investigation of phytochemicals from *Asparagus racemosus* as plausible antiviral agent in COVID-19. J. Biomol. Struct. Dyn., 1–15 .10.1080/07391102.2020.1784289 32579064

[B24] Daak A. A. Lopez-Toledano M. A. Heeney M. M. (2020). Biochemical and therapeutic effects of omega-3 fatty acids in sickle cell disease. Complement. Therapies Med. 52. 102482. 10.1016/j.ctim.2020.102482 32951732

[B25] de Kloet A. D. Krause E. G. Woods S. C. (2010). The renin angiotensin system and the metabolic syndrome. Physiol. Behav. 100 (5), 525–534. 10.1016/j.physbeh.2010.03.018 20381510 PMC2886177

[B26] de Wit E. van Doremalen N. Falzarano D. Munster V. J. (2016). SARS and MERS: recent insights into emerging coronaviruses. Nat. Rev. Microbiol. 14 (8), 523–534. 10.1038/nrmicro.2016.81 27344959 PMC7097822

[B28] Donoghue M. Hsieh F. Baronas E. Godbout K. Gosselin M. Stagliano N. (2000). A novel angiotensin-converting enzyme-related carboxypeptidase (ACE2) converts angiotensin I to angiotensin 1-9. Circ. Res. 87 (5), e1–e9. 10.1161/01.res.87.5.e1 10969042

[B29] Enmozhi S. K. Raja K. Sebastine I. Joseph J. (2020). Andrographolide as a potential inhibitor of SARS-CoV-2 main protease: an in silico approach. J. Biomol. Struct. Dyn. 1-7 10.1080/07391102.2020.1760136 PMC7212536 32329419

[B30] Esparza T. J. Martin N. P. Anderson G. P. Goldman E. R. Brody D. L. (2020). High affinity nanobodies block SARS-CoV-2 spike receptor binding domain interaction with human angiotensin converting enzyme. Sci. Rep. 10 (1), 22370. 10.1038/s41598-020-79036-0 33353972 PMC7755911

[B31] European Centre for Disease Prevention and Control (2020). COVID-19 situation update worldwide, as of 26 September 2020 Available from: https://www.ecdc.europa.eu/en/geographical-distribution-2019-ncov-cases (Accessed September 26, 2020).

[B32] Fan C. Li K. Ding Y. Lu W. L. Wang J. (2020). ACE2 expression in kidney and testis may cause kidney and testis damage after 2019-nCoV infection. medRxiv 10.1101/2020.02.12.20022418 PMC7838217 33521006

[B33] Fougeroux C. Goksøyr L. Idorn M. Soroka V. Myeni S. K. Dagil R. (2021). Capsid-like particles decorated with the SARS-CoV-2 receptor-binding domain elicit strong virus neutralization activity. Nat. Commun. 12 (1), 324. 10.1038/s41467-020-20251-8 33436573 PMC7804149

[B34] Furuhashi M. Moniwa N. Takizawa H. Ura N. Shimamoto K. (2020). Potential differential effects of renin-angiotensin system inhibitors on SARS-CoV-2 infection and lung injury in COVID-19. Hypertens. Res. 43 (8), 837–840. 10.1038/s41440-020-0478-1 32433641 PMC7237878

[B35] Gheblawi M. Wang K. Viveiros A. Nguyen Q. Zhong J.-C. Turner A. J. (2020). Angiotensin-converting enzyme 2: SARS-CoV-2 receptor and regulator of the renin-angiotensin system. Circ. Res. 126 (10), 1456–1474. 10.1161/circresaha.120.317015 32264791 PMC7188049

[B36] Glasgow A. Glasgow J. Limonta D. Solomon P. Lui I. Zhang Y. (2020). Engineered ACE2 receptor traps potently neutralize SARS-CoV-2. Proc. Natl. Acad. Sci. USA 117 (45), 28046. 10.1073/pnas.2016093117 33093202 PMC7668070

[B37] Guan W.-j. Ni Z.-y. Hu Y. Liang W.-h. Ou C.-q. He J.-x. (2020). Clinical characteristics of coronavirus disease 2019 in China. N. Engl. J. Med. 382 (18), 1708–1720. 10.1056/nejmoa2002032 32109013 PMC7092819

[B38] Güler H. I. Tatar G. Yildiz O. Belduz A. O. Kolayli S. (2020). Investigation of potential inhibitor properties of ethanolic propolis extracts against ACE-II receptors for COVID-19 treatment by Molecular Docking Study. ScienceOpen Preprints. 10.35206/jan.762734 PMC8098016 33950349

[B39] Gupta R. Misra A. (2020). Contentious issues and evolving concepts in the clinical presentation and management of patients with COVID-19 infectionwith reference to use of therapeutic and other drugs used in Co-morbid diseases (Hypertension, diabetes etc). Diabetes Metab. Syndr. Clin. Res. Rev. 14 (3), 251–254. 10.1016/j.dsx.2020.03.012 PMC7102586 32247213

[B40] Gupte M. (2011). Role of angiotensin converting enzyme 2 (ACE2) in obesity -associated hypertension. Lexington, Kentucky: University of Kentucky Doctoral Dissertations Available from: https://uknowledge.uky.edu/gradschool_diss/37 (Accessed October 28, 2020).

[B41] Gutekunst W. R. Gianatassio R. Baran P. S. (2012). Sequential C sp 3‐H arylation and olefination: total synthesis of the proposed structure of pipercyclobutanamide A. Angew. Chem. Int. Ed. 51 (30), 7507–7510. 10.1002/anie.201203897 PMC3495593 22715157

[B42] Gutierrez-Villagomez J. M. Campos-García T. Molina-Torres J. López M. G. Vázquez-Martínez J. (2020). Alkamides and piperamides as potential antivirals against the severe acute respiratory syndrome coronavirus 2 (SARS-CoV-2). J. Phys. Chem. Lett. 11 (19), 8008–8016. 10.1021/acs.jpclett.0c01685 32840378

[B43] Haggag Y. A. El-Ashmawy N. E. Okasha K. M. (2020). Is hesperidin essential for prophylaxis and treatment of COVID-19 Infection?. Med. Hypotheses 144, 109957. 10.1016/j.mehy.2020.109957 32531538 PMC7274964

[B44] Hamming I. Timens W. Bulthuis M. Lely A. Navis G. van Goor H. (2004). Tissue distribution of ACE2 protein, the functional receptor for SARS coronavirus. A first step in understanding SARS pathogenesis. J. Pathol. 203 (2), 631–637. 10.1002/path.1570 15141377 PMC7167720

[B45] Hao Y. Liu Y. (2016). Osthole alleviates bleomycin-induced pulmonary fibrosis via modulating angiotensin-converting enzyme 2/angiotensin-(1-7) *Axis* and decreasing inflammation responses in rats. Biol. Pharm. Bull. 39 (4), 457–465. 10.1248/bpb.b15-00358 26822530

[B46] Haschke M. Schuster M. Poglitsch M. Loibner H. Salzberg M. Bruggisser M. (2013). Pharmacokinetics and pharmacodynamics of recombinant human angiotensin-converting enzyme 2 in healthy human subjects. Clin. Pharmacokinet. 52 (9), 783–792. 10.1007/s40262-013-0072-7 23681967

[B48] He R. Yang Y.-J. Wang Z. Xing C.-R. Yuan J. Wang L.-F. (2019). Rapeseed protein-derived peptides, LY, RALP, and GHS, modulates key enzymes and intermediate products of renin-angiotensin system pathway in spontaneously hypertensive rat. NPJ Sci. Food 3, 1. 10.1038/s41538-018-0033-5 31304273 PMC6550218

[B49] Hellinger R. Gruber C. W. (2019). Peptide-based protease inhibitors from plants. Drug Discov. Today 24 (9), 1877–1889. 10.1016/j.drudis.2019.05.026 31170506 PMC6753016

[B50] Ho T. Wu S. Chen J. Li C. Hsiang C. (2007). Emodin blocks the SARS coronavirus spike protein and angiotensin-converting enzyme 2 interaction. Antivir. Res. 74 (2), 92–101. 10.1016/j.antiviral.2006.04.014 16730806 PMC7114332

[B51] Hoffmann M. Kleine-Weber H. Schroeder S. Krüger N. Herrler T. Erichsen S. (2020). SARS-CoV-2 cell entry depends on ACE2 and TMPRSS2 and is blocked by a clinically proven protease inhibitor. Cell 181 (2), 271–280. 10.1016/j.cell.2020.02.052 32142651 PMC7102627

[B53] Huang C. Wang Y. Li X. Ren L. Zhao J. Hu Y. (2020). Clinical features of patients infected with 2019 novel coronavirus in Wuhan, China. The Lancet 395 (10223), 497–506. 10.1016/s0140-6736(20)30183-5 PMC7159299 31986264

[B54] Imai Y. Kuba K. Rao S. Huan Y. Guo F. Guan B. (2005). Angiotensin-converting enzyme 2 protects from severe acute lung failure. Nature 436 (7047), 112–116. 10.1038/nature03712 16001071 PMC7094998

[B55] Joshi T. Joshi T. Sharma P. Mathpal S. Pundir H. Bhatt V. (2020). In silico screening of natural compounds against COVID-19 by targeting Mpro and ACE2 using molecular docking. Eur. Rev. Med. Pharmacol. Sci. 24 (8), 4529–4536. 10.26355/eurrev_202004_21036 32373991

[B56] Kai H. Kai M. (2020). Interactions of coronaviruses with ACE2, angiotensin II, and RAS inhibitors-lessons from available evidence and insights into COVID-19. Hypertens. Res. 43 (7), 648–654. 10.1038/s41440-020-0455-8 32341442 PMC7184165

[B57] Khan A. Benthin C. Zeno B. Albertson T. E. Boyd J. Christie J. D. (2017). A pilot clinical trial of recombinant human angiotensin-converting enzyme 2 in acute respiratory distress syndrome. Crit. Care (London, England) 21 (1), 234. 10.1186/s13054-017-1823-x PMC5588692 28877748

[B58] Kuba K. Imai Y. Rao S. Gao H. Guo F. Guan B. (2005). A crucial role of angiotensin converting enzyme 2 (ACE2) in SARS coronavirus-induced lung injury. Nat. Med. 11 (8), 875–879. 10.1038/nm1267 16007097 PMC7095783

[B59] Kumar B. K. Sekhar K. V. G. C. Kunjiappan S. Jamalis J. Balaña-Fouce R. Tekwani B. L. (2020a). Druggable targets of SARS-CoV-2 and treatment opportunities for COVID-19. Bioorg. Chem. 104,.104269. 10.1016/j.bioorg.2020.104269 32947136 PMC7476961

[B60] Kumar S. Nyodu R. Maurya V. K. Saxena S. K. (2020b). “Morphology, genome organization, replication, and pathogenesis of severe acute respiratory syndrome coronavirus 2 (SARS-CoV-2),” in Coronavirus disease 2019 (COVID-19): epidemiology, pathogenesis, diagnosis, and therapeutics. Editor Saxena S. K. (Singapore: Springer Singapore), 23–31.

[B61] Kumar V. Dhanjal J. K. Bhargava P. Kaul A. Wang J. Zhang H. (2020c). Withanone and Withaferin-A are predicted to interact with transmembrane protease serine 2 (TMPRSS2) and block entry of SARS-CoV-2 into cells. J. Biomol. Struct. Dyn., 1–13. 10.1080/07391102.2020.1775704 PMC7309304 32469279

[B62] Lalaoui R. Bakour S. Raoult D. Verger P. Sokhna C. Devaux C. (2020). What could explain the late emergence of COVID-19 in Africa?. New Microbes New Infect. 38, 100760. 10.1016/j.nmni.2020.100760 32983542 PMC7508045

[B63] Letko M. Marzi A. Munster V. (2020). Functional assessment of cell entry and receptor usage for SARS-CoV-2 and other lineage B betacoronaviruses. Nat. Microbiol. 5 (4), 562–569. 10.1038/s41564-020-0688-y 32094589 PMC7095430

[B64] Li M. Y. Li L. Zhang Y. Wang X. S. (2020a). Expression of the SARS-CoV-2 cell receptor gene ACE2 in a wide variety of human tissues. Infect. Dis. poverty 9 (1), 45. 10.1186/s40249-020-00662-x 32345362 PMC7186534

[B65] Li Y. C. Bai W. Z. Hashikawa T. (2020b). The neuroinvasive potential of SARS‐CoV2 may play a role in the respiratory failure of COVID‐19 patients. J. Med. Virol. 92 (6), 552–555. 10.1002/jmv.25728 32104915 PMC7228394

[B66] Li Z. Wu M. Guo J. Yao J. Liao X. Song S. (2020c). Caution on kidney dysfunctions of 2019-nCoV patients. MedRxiv. 10.1101/2020.02.08.20021212

[B67] Liao W. Fan H. Davidge S. T. Wu J. (2019). Egg white-derived antihypertensive peptide IRW (Ile-Arg-Trp) reduces blood pressure in spontaneously hypertensive rats via the ACE2/ang (1-7)/mas receptor *Axis* . Mol. Nutr. Food Res. 63 (9), e1900063. 10.1002/mnfr.201900063 30913349 PMC6594022

[B68] Liao W. (2019). Food protein-derived peptides targeting angiotensin converting enzyme 2. Education and research archive (ERA). Edmonton, Alberta: University of Alberta (Accessed October 15, 2020). 10.7939/r3-4v2n-7x45

[B69] Lippi G. Lavie C. J. Henry B. M. Sanchis-Gomar F. (2020). Do genetic polymorphisms in angiotensin converting enzyme 2 (ACE2) gene play a role in coronavirus disease 2019 (COVID-19)? Clin. Chem. Lab. Med. (Cclm), 1 10.1515/cclm-2020-0727 32598305

[B70] Liu H. Jiang Y. Li M. Yu X. Sui D. Fu L. (2019). Ginsenoside Rg3 attenuates angiotensin II-mediated renal injury in rats and mice by upregulating angiotensin-converting enzyme 2 in the renal tissue. Evidence-Based Complement. Altern. Med. 10.1155/2019/6741057 PMC6915024 31885658

[B71] Liu R. Zhang M. Wyche T. P. Winston-McPherson G. N. Bugni T. S. Tang W. (2012). Stereoselective preparation of cyclobutanes with four different substituents: total synthesis and structural revision of pipercyclobutanamide A and piperchabamide G. Angew. Chem. Int. Ed. 51 (30), 7503–7506. 10.1002/anie.201203379 PMC3444511 22715150

[B73] Majumdar S. Mohanta B. C. Chowdhury D. R. Banik R. Dinda B. Basak A. (2010). Proprotein convertase inhibitory activities of flavonoids isolated from Oroxylum indicum. Cmc 17 (19), 2049–2058. 10.2174/092986710791233643 20423311

[B74] Majumder K. Liang G. Chen Y. Guan L. Davidge S. T. Wu J. (2015). Egg ovotransferrin‐derived ACE inhibitory peptide IRW increases ACE2 but decreases proinflammatory genes expression in mesenteric artery of spontaneously hypertensive rats. Mol. Nutr. Food Res. 59 (9), 1735–1744. 10.1002/mnfr.201500050 26016560 PMC5034750

[B75] Malami I. Abdul A. B. Abdullah R. Bt Kassim N. K. Waziri P. Christopher Etti I. (2016). Silico discovery of potential uridine-cytidine kinase 2 inhibitors from the rhizome of alpinia mutica, Molecules (Basel, Switzerland),21, 417 10.3390/molecules21040417 PMC6274218 27070566

[B76] Marian A. J. (2013). The discovery of the ACE2 gene. Circ. Res. 112 (10), 1307–1309. 10.1161/circresaha.113.301271 23661710

[B78] Monteil V. Kwon H. Prado P. Hagelkrüys A. Wimmer R. A. Stahl M. (2020). Inhibition of SARS-CoV-2 infections in engineered human tissues using clinical-grade soluble human ACE2. Cell 181 (4), 905–913. 10.1016/j.cell.2020.04.004 32333836 PMC7181998

[B79] Moyo M. Aremu A. O. Van Staden J. (2015). Medicinal plants: an invaluable, dwindling resource in sub-Saharan Africa. J. Ethnopharmacology 174, 595–606. 10.1016/j.jep.2015.04.034 25929451

[B80] Muhammad A. Mada S. B. Malami. I. Forcados G. E. Erukainure O. L. Sani H. (2018). Postmenopausal osteoporosis and breast cancer: the biochemical links and beneficial effects of functional foods. Biomed. Pharmacother. = Biomedecine pharmacotherapie 107, 571–582 . 10.1016/j.biopha.2018.08.018 30114641

[B81] My T. T. A. Loan H. T. P. Hai N. T. T. Hieu L. T. Hoa T. T. Thuy B. T. P. (2020). Evaluation of the inhibitory activities of COVID‐19 of Melaleuca cajuputi oil using docking simulation. ChemistrySelect 5 (21), 6312–6320. 10.1002/slct.202000822 32572383 PMC7300966

[B82] Naqvi A. A. T. Fatima K. Mohammad T. Fatima U. Singh I. K. Singh A. (2020). Insights into SARS-CoV-2 genome, structure, evolution, pathogenesis and therapies: structural genomics approach. Biochim. Biophys. Acta (Bba) - Mol. Basis Dis. 1866 (10), 165878. 10.1016/j.bbadis.2020.165878 PMC7293463 32544429

[B83] Ngwa W. Kumar R. Thompson D. Lyerly W. Moore R. Reid T.-E. (2020). Potential of flavonoid-inspired phytomedicines against COVID-19. Molecules 25 (11), 2707. 10.3390/molecules25112707 PMC7321405 32545268

[B84] Nicoletti M. (2012). Nutraceuticals and botanicals: overview and perspectives. Int. J. Food Sci. Nutr. 63 (Suppl. 1 2)–6. 10.3109/09637486.2011.628012 22360273

[B86] Nittari G. Pallotta G. Amenta F. Tayebati S. K. (2020). Current pharmacological treatments for SARS-CoV-2: a narrative review. Eur. J. Pharmacol. 882, 173328. 10.1016/j.ejphar.2020.173328 32603692 PMC7320862

[B87] Ohashi H. Watashi K. Saso W. Shionoya K. Iwanami S. Hirokawa T. (2020). Multidrug treatment with nelfinavir and cepharanthine against COVID-19. bioRxiv 10.1101/2020.04.14.039925

[B88] Okasha K. M. (2020). Hesperidin and diosmin for treatment of COVID-19. Available from: https://clinicaltrials.gov/show/NCT04452799 (Accessed October 28, 2020). 10.1016/j.mehy.2020.109957 PMC7274964 32531538

[B89] Omar S. Bouziane I. Bouslama Z. Djemel A. (2020). (ACE2) from natural products: quercetin, hispidulin, and cirsimaritin exhibited better potential inhibition than hydroxy-chloroquine against COVID-19 main protease active site and ACE2. Chemrxiv. Preprint.In-silico identification of potent inhibitors of COVID-19 main protease (Mpro) and angiotensin converting enzyme 2 10.26434/chemrxiv.12181404.v1

[B90] Omotuyi I. O. Nash O. Ajiboye B. O. Olumekun V. O. Oyinloye B. E. Osuntokun O. T. (2020). Aframomum melegueta K.Schum. secondary metabolites exhibit polypharmacology against SARS-CoV-2 drug targets: *in vitro* validation of furin inhibition.Phytotherapy research : ptr 10.1002/ptr.6843 32964551

[B91] Oyebode O. Kandala N.-B. Chilton P. J. Lilford R. J. (2016). Use of traditional medicine in middle-income countries: a WHO-SAGE study. Health Policy Plan. 31 (8), 984–991. 10.1093/heapol/czw022 27033366 PMC5013777

[B92] Palau V. Riera M. Soler M. J. (2020). ADAM17 inhibition may exert a protective effect on COVID-19. Nephrol. Dial. Transpl. 35 (6), 1071–1072. 10.1093/ndt/gfaa093 PMC7184459 32291449

[B93] Paraiso I. L. Revel J. S. Stevens J. F. (2020). Potential use of polyphenols in the battle against COVID-19. Curr. Opin. Food Sci. 32, 149. 10.1016/j.cofs.2020.08.004 32923374 PMC7480644

[B94] Peiris J. S. M. Yuen K. Y. Osterhaus A. D. M. E. Stöhr K. (2003). The severe acute respiratory syndrome. N. Engl. J. Med. 349 (25), 2431–2441. 10.1056/nejmra032498 14681510

[B95] Poochi S. P. Easwaran M. Balasubramanian B. Anbuselvam M. Meyyazhagan A. Park S. (2020). Employing bioactive compounds derived from *Ipomoea* obscura (L.) to evaluate potential inhibitor for SARS-CoV-2 main protease and ACE2 protein. Food Front. 10.1002/fft2.29 PMC7361879 32838301

[B96] Rakib A. Paul A. Chy M. N. U. Sami S. A. Baral S. K. Majumder M. (2020). Biochemical and computational approach of selected phytocompounds from Tinospora crispa in the management of COVID-19. Molecules 25 (17), 3936. 10.3390/molecules25173936 PMC7504753 32872217

[B97] Rattanapisit K. Shanmugaraj B. Manopwisedjaroen S. Purwono P. B. Siriwattananon K. Khorattanakulchai N. (2020). Rapid production of SARS-CoV-2 receptor binding domain (RBD) and spike specific monoclonal antibody CR3022 in Nicotiana benthamiana. Sci. Rep. 10 (1), 17698. 10.1038/s41598-020-74904-1 33077899 PMC7573609

[B98] Ren X. Shao X.-X. Li X.-X. Jia X.-H. Song T. Zhou W.-Y. (2020). Identifying potential treatments of COVID-19 from Traditional Chinese Medicine (TCM) by using a data-driven approach. J. Ethnopharmacology 258, 112932. 10.1016/j.jep.2020.112932 PMC7196535 32376368

[B99] Sakshi C. Harikrishnan A. Jayaraman S. Choudhury A. R. Veena V. (2021). Predictive medicinal metabolites from Momordica dioica against comorbidity related proteins of SARS-CoV-2 infections. J. Biomol. Struct. Dyn., 1–14. 10.1080/07391102.2020.1868340 PMC7814569 33427588

[B100] Senthil Kumar K. J. Gokila Vani M. Wang C. S. Chen C. C. Chen Y. C. Lu L. P. (2020). *Geranium* and lemon essential oils and their active compounds downregulate angiotensin-converting enzyme 2 (ACE2), a SARS-CoV-2 spike receptor-binding domain, Plants (Basel, Switzerland) Epithelial cells, 9 10.3390/plants9060770 PMC7355681 32575476

[B101] Shah V. K. Firmal P. Alam A. Ganguly D. Chattopadhyay S. (2020). Overview of immune response during SARS-CoV-2 infection: lessons from the past. Front. Immunol. 11. 10.3389/fimmu.2020.01949 PMC7426442 32849654

[B102] Shang J. Wan Y. Luo C. Ye G. Geng Q. Auerbach A. (2020). Cell entry mechanisms of SARS-CoV-2. Proc. Natl. Acad. Sci. USA 117 (21), 11727–11734. 10.1073/pnas.2003138117 32376634 PMC7260975

[B103] Shi X. Gong E. Gao D. Zhang B. Zheng J. Gao Z. (2005). Severe acute respiratory syndrome associated coronavirus is detected in intestinal tissues of fatal cases. Am. J. Gastroenterol. 100 (1), 169–176. 10.1111/j.1572-0241.2005.40377.x 15654797

[B104] Shi Y. Zhang B. Chen X.-J. Xu D.-Q. Wang Y.-X. Dong H.-Y. (2013). Osthole protects lipopolysaccharide-induced acute lung injury in mice by preventing down-regulation of angiotensin-converting enzyme 2. Eur. J. Pharm. Sci. 48 (4-5), 819–824. 10.1016/j.ejps.2012.12.031 23321685

[B105] Shil P. K. Kwon K.-C. Zhu P. Verma A. Daniell H. Li Q. (2014). Oral delivery of ACE2/Ang-(1-7) bioencapsulated in plant cells protects against experimental uveitis and autoimmune uveoretinitis. Mol. Ther. 22 (12), 2069–2082. 10.1038/mt.2014.179 25228068 PMC4429699

[B106] Sinha S. K. Prasad S. K. Islam M. A. Gurav S. S. Patil R. B. AlFaris N. A. (2020a). Identification of bioactive compounds from *Glycyrrhiza* glabra as possible inhibitor of SARS-CoV-2 spike glycoprotein and non-structural protein-15: a pharmacoinformatics study. J. Biomol. Struct. Dyn., 1–15. 10.1080/07391102.2020.1779132 PMC7309308 32552462

[B107] Sinha S. K. Shakya A. Prasad S. K. Singh S. Gurav N. S. Prasad R. S. (2020b). An in-silico evaluation of different Saikosaponins for their potency against SARS-CoV-2 using NSP15 and fusion spike glycoprotein as targets. J. Biomol. Struct. Dyn., 1–12. 10.1080/07391102.2020.1762741 PMC7232888 32345124

[B108] Sui H. Yu Q. Zhi Y. Geng G. Liu H. Xu H. (2010). [Effects of apigenin on the expression of angiotensin-converting enzyme 2 in kidney in spontaneously hypertensive rats]. Wei Sheng Yan Jiu 39 (6), 693–700. 21351633

[B109] Sun T. Guan J. (2020). Novel coronavirus and the central nervous system. Eur. J. Neurol. 10.1111/ene.14227 32216009

[B110] Takahashi S. Yoshiya T. Yoshizawa-Kumagaye K. Sugiyama T. (2015). Nicotianamine is a novel angiotensin-converting enzyme 2 inhibitor in soybean. Biomed. Res. 36 (3), 219–224. 10.2220/biomedres.36.219 26106051

[B111] Tan T. K. Rijal P. Rahikainen R. Keeble A. H. Schimanski L. Hussain S. (2021). A COVID-19 vaccine candidate using SpyCatcher multimerization of the SARS-CoV-2 spike protein receptor-binding domain induces potent neutralising antibody responses. Nat. Commun. 12 (1), 542. 10.1038/s41467-020-20654-7 33483491 PMC7822889

[B112] Thuy B. T. P. My T. T. A. Hai N. T. T. Hieu L. T. Hoa T. T. Thi Phuong Loan H. (2020). Investigation into SARS-CoV-2 resistance of compounds in garlic essential oil. ACS omega 5 (14), 8312–8320. 10.1021/acsomega.0c00772 32363255 PMC7123907

[B113] Tikellis C. Thomas M. C. (2012). Angiotensin-converting enzyme 2 (ACE2) is a key modulator of the renin angiotensin system in health and disease. Int. J. Pept. 2012, 256294. 10.1155/2012/256294 22536270 PMC3321295

[B114] To K. Tong J. H. Chan P. K. Au F. W. Chim S. S. Allen Chan K. (2004). Tissue and cellular tropism of the coronavirus associated with severe acute respiratory syndrome: an *in-situ* hybridization study of fatal cases. J. Pathol. 202 (2), 157–163. 10.1002/path.1510 14743497 PMC7167900

[B115] Turner A. J. Tipnis S. R. Guy J. L. Rice G. I. Hooper N. M. (2002). ACEH/ACE2 is a novel mammalian metallocarboxypeptidase and a homologue of angiotensin-converting enzyme insensitive to ACE inhibitors. Can. J. Physiol. Pharmacol. 80 (4), 346–353. 10.1139/y02-021 12025971

[B116] Tutunchi H. Naeini F. Ostadrahimi A. Hosseinzadeh‐Attar M. J. (2020). Naringenin, a flavanone with antiviral and anti‐inflammatory effects: a promising treatment strategy against COVID ‐19. Phytotherapy Res. 34, 3137. 10.1002/ptr.6781 PMC7361426 32613637

[B117] Ulu A. Harris T. R. Morisseau C. Miyabe C. Inoue H. Schuster G. (2013). Anti-inflammatory effects of ω-3 polyunsaturated fatty acids and soluble epoxide hydrolase inhibitors in angiotensin-II-dependent hypertension. J. Cardiovasc. Pharmacol. 62 (3), 285–297. 10.1097/fjc.0b013e318298e460 23676336 PMC3773051

[B118] Utomo R. Y. Ikawati M. Meiyanto E. (2020). Revealing the potency of citrus and galangal constituents to halt SARS-CoV-2 infection. Preprints, 2020030214. 10.20944/preprints202003.0214.v1

[B120] Verdecchia P. Cavallini C. Spanevello A. Angeli F. (2020). The pivotal link between ACE2 deficiency and SARS-CoV-2 infection. Eur. J. Intern. Med. 76, 14–20. 10.1016/j.ejim.2020.04.037 32336612 PMC7167588

[B121] Wahedi H. M. Ahmad S. Abbasi S. W. (2020). Stilbene-based natural compounds as promising drug candidates against COVID-19. J. Biomol. Struct. Dyn. 1-10 10.1080/07391102.2020.1762743 32345140

[B123] Wang J. Zhao S. Liu M. Zhao Z. Xu Y. Wang P. (2020a). ACE2 expression by colonic epithelial cells is associated with viral infection, immunity and energy metabolism. medRxiv. 10.1101/2020.02.05.20020545 PMC12891528 41554833

[B124] Wang Q. Qiu Y. Li J.-Y. Zhou Z.-J. Liao C.-H. Ge X.-Y. (2020b). A unique protease cleavage site predicted in the spike protein of the novel pneumonia coronavirus (2019-nCoV) potentially related to viral transmissibility. Virol. Sin. 35 (3), 337–339. 10.1007/s12250-020-00212-7 32198713 PMC7091172

[B125] Wang S. Qiu Z. Hou Y. Deng X. Xu W. Zheng T. (2021). AXL is a candidate receptor for SARS-CoV-2 that promotes infection of pulmonary and bronchial epithelial cells. Cel Res. 10.1038/s41422-020-00460-y PMC7791157 33420426

[B126] Wang Z. Wang S. Zhao J. Yu C. Hu Y. Tu Y. (2019). Naringenin ameliorates renovascular hypertensive renal damage by normalizing the balance of renin-angiotensin system components in rats. Int. J. Med. Sci. 16 (5), 644–653. 10.7150/ijms.31075 31217731 PMC6566737

[B127] Watanabe Y. Allen J. D. Wrapp D. McLellan J. S. Crispin M. (2020). Site-specific glycan analysis of the SARS-CoV-2 spike. Science (New York, N.Y.) 369 (6501), 330–333 . 10.1126/science.abb9983 PMC7199903 32366695

[B128] Wei X. Zhu X. Hu N. Zhang X. Sun T. Xu J. (2015). Baicalin attenuates angiotensin II-induced endothelial dysfunction. Biochem. Biophysical Res. Commun. 465 (1), 101–107. 10.1016/j.bbrc.2015.07.138 26239661

[B129] WHO (2004). Summary of probable SARS cases with onset of illness from 1 November 2002 to 31 July 2003 (Based on data as of the 31 December 2003) World Health Organisation (WHO). Available from: https://www.who.int/csr/sars/country/table2004_04_21/en/ (Accessed September 30, 2020).

[B130] WHO (2019). WHO global report on traditional and complimentary medicine 2019. Geneva: World Health Organization Available from: https://apps.who.int/iris/handle/10665/312342 (Accessed.September 30, 2020).

[B131] Williamson G. Kerimi A. (2020). Testing of natural products in clinical trials targeting the SARS-CoV-2 (Covid-19) viral spike protein-angiotensin converting enzyme-2 (ACE2) interaction. Biochem. Pharmacol. 178, 114123. 10.1016/j.bcp.2020.114123 32593613 PMC7316054

[B134] Wu Y. Xu X. Chen Z. Duan J. Hashimoto K. Yang L. (2020). Nervous system involvement after infection with COVID-19 and other coronaviruses. Brain Behav. Immun. 87, 18–22. 10.1016/j.bbi.2020.03.031 32240762 PMC7146689

[B135] Yahalom-Ronen Y. Tamir H. Melamed S. Politi B. Shifman O. Achdout H. (2020). A single dose of recombinant VSV-∆G-spike vaccine provides protection against SARS-CoV-2 challenge. Nat. Commun. 11 (1), 6402. 10.1038/s41467-020-20228-7 33328475 PMC7745033

[B136] Yang L. Li Y.-T. Miao J. Wang L. Fu H. Li Q. (2020a). Network pharmacology studies on the effect of Chai-Ling decoction in coronavirus disease 2019. Traditional Med. Res. 5 (3), 145–159.

[B137] Yang X. Yu Y. Xu J. Shu H. Xia J. a. Liu H. (2020b). Clinical course and outcomes of critically ill patients with SARS-CoV-2 pneumonia in Wuhan, China: a single-centered, retrospective, observational study. Lancet Respir. Med. 8 (5), 475–481. 10.1016/s2213-2600(20)30079-5 32105632 PMC7102538

[B138] Yu L. Yuan K. Phuong H. T. A. Park B. M. Kim S. H. (2016). Angiotensin-(1-5), an active mediator of renin-angiotensin system, stimulates ANP secretion via Mas receptor. Peptides 86, 33–41. 10.1016/j.peptides.2016.09.009 27660028

[B140] Zhang H. Penninger J. M. Li Y. Zhong N. Slutsky A. S. (2020a). Angiotensin-converting enzyme 2 (ACE2) as a SARS-CoV-2 receptor: molecular mechanisms and potential therapeutic target. Intensive Care Med. 46 (4), 586–590. 10.1007/s00134-020-05985-9 32125455 PMC7079879

[B141] Zhang Y. Geng X. Tan Y. Li Q. Xu C. Xu J. (2020b). New understanding of the damage of SARS-CoV-2 infection outside the respiratory system. Biomed. Pharmacother. 127, 110195. 10.1016/j.biopha.2020.110195 32361161 PMC7186209

[B142] Zhao Y. Zhao Z. Wang Y. Zhou Y. Ma Y. Zuo W. (2020). Single-cell RNA expression profiling of ACE2, the receptor of SARS-CoV-2. bioRxiv . 10.1101/2020.01.26.919985 PMC7462411 32663409

[B143] Zheng J. Wang J. Pan H. Wu H. Ren D. Lu J. (2017). Effects of IQP, VEP and Spirulina platensis hydrolysates on the local kidney renin angiotensin system in spontaneously hypertensive rats. Mol. Med. Rep. 16 (6), 8485–8492. 10.3892/mmr.2017.7602 28944898

[B144] Zhou P. Yang X.-L. Wang X.-G. Hu B. Zhang L. Zhang W. (2020). A pneumonia outbreak associated with a new coronavirus of probable bat origin. Nature 579 (7798), 270–273. 10.1038/s41586-020-2012-7 32015507 PMC7095418

[B145] Zi C.-T. Zhang N. Yang L. Wang L.-X. Wu Y.-L. Su Y.-S. (2020). Discovery of a potent angiotensin converting enzyme 2 inhibitor from Chinese medicinal and edible plant via docking-based virtual screening. Research square. 10.21203/rs.3.rs-32515/v1

